# Alzheimer’s disease and neuroinflammation: will new drugs in clinical trials pave the way to a multi-target therapy?

**DOI:** 10.3389/fphar.2023.1196413

**Published:** 2023-06-02

**Authors:** Daniela Melchiorri, Sara Merlo, Benjamin Micallef, John-Joseph Borg, František Dráfi

**Affiliations:** ^1^ Department of Physiology and Pharmacology, Sapienza University, Rome, Italy; ^2^ Department of Biomedical and Biotechnological Sciences, Section of Pharmacology, University of Catania, Catania, Italy; ^3^ Malta Medicines Authority, San Ġwann, Malta; ^4^ School of Pharmacy, Department of Biology, University of Tor Vergata, Rome, Italy; ^5^ Institute of Experimental Pharmacology and Toxicology, Centre of Experimental Medicine SAS Bratislava, Bratislava, Slovakia; ^6^ State Institute for Drug Control, Bratislava, Slovakia

**Keywords:** Alzhaimer’s disease (AD), amyloid-beta, microglia, astrocyte, multi-target therapy, neuroinflmamation

## Abstract

Despite extensive research, no disease-modifying therapeutic option, able to prevent, cure or halt the progression of Alzheimer’s disease [AD], is currently available. AD, a devastating neurodegenerative pathology leading to dementia and death, is characterized by two pathological hallmarks, the extracellular deposits of amyloid beta (Aβ) and the intraneuronal deposits of neurofibrillary tangles (NFTs) consisting of altered hyperphosphorylated tau protein. Both have been widely studied and pharmacologically targeted for many years, without significant therapeutic results. In 2022, positive data on two monoclonal antibodies targeting Aβ, donanemab and lecanemab, followed by the 2023 FDA accelerated approval of lecanemab and the publication of the final results of the phase III Clarity AD study, have strengthened the hypothesis of a causal role of Aβ in the pathogenesis of AD. However, the magnitude of the clinical effect elicited by the two drugs is limited, suggesting that additional pathological mechanisms may contribute to the disease. Cumulative studies have shown inflammation as one of the main contributors to the pathogenesis of AD, leading to the recognition of a specific role of neuroinflammation synergic with the Aβ and NFTs cascades. The present review provides an overview of the investigational drugs targeting neuroinflammation that are currently in clinical trials. Moreover, their mechanisms of action, their positioning in the pathological cascade of events that occur in the brain throughout AD disease and their potential benefit/limitation in the therapeutic strategy in AD are discussed and highlighted as well. In addition, the latest patent requests for inflammation-targeting therapeutics to be developed in AD will also be discussed.

## 1 Introduction

The worldwide prevalence of people with dementia is forecasted to triple in 2050 compared to 2019, reaching 150 million of cases (GBD, 2019 Dementia Forecasting Collaborators, 2022). AD is the most common type of dementia, accounting for 60%–70% of all dementia cases (Huang L. K. et al., 2020), and is at present without a cure. Alzheimer’s disease was officially listed as the sixth-leading cause of death in the United States in 2019 (National Center for Health Statistics. 2020-2021). Consequently, healthcare and long-term care costs for individuals with Alzheimer’s or other dementias are substantial. Dementia is one of the costliest conditions to the society (Hurd et al., 2013). Support and care for AD patients is often on families, and the caregiver burden is huge (Bailes et al., 2016). According to recent understanding of the pathophysiology of AD, the disease develops as a continuum including: i. a preclinical stage, characterized by measurable brain changes but no symptoms; ii**.** a prodromal stage, also termed mild cognitive impairment (MCI) due to AD, encompassing both changes in the brain as well as very mild clinical signs that may not interfere with patient’s functioning, and iii. dementia due to AD. The length of each part of the continuum is influenced by age, genetics, biological sex and other factors and thus is significantly variable across individuals (Vermunt et al., 2019). Among the total population of patients with MCI, the annual conversion rate to dementia due to AD is estimated to be 10%–15% (Liu et al., 2013). However, some individuals with MCI do not further progress in the disease or even revert to normal cognition. The current lack of a comprehensive knowledge on the different factors that orientate the trajectory of patients in the preclinical and prodromal AD stages challenges the design of both disease-modifying drugs as well as clinical trials testing potential new drugs.

In the past few decades research in AD focused mainly on the deposition in the brain of the protein fragment beta-amyloid (Aβ) into extracellular plaques followed by the accumulation of an abnormal, hyperphosphorylated form of the protein tau (p-tau), as the main driving factors in the AD pathology, leading to extended neuronal damage and synaptic dysfunction. The so-called Aβ hypothesis of AD is mainly supported by genetic data as all known mutations linked to familial AD affect the production or aggregation tendency of Aβ, and AD-protective mutation variants of the Aβ precursor protein, APP, have been reported (Jonsson et al., 2012). Both Aβ and p-tau have thus been selected as potential biomarkers to help identifying MCI patients more likely to progress to AD dementia, and monoclonal antibodies targeting different forms of Aβ have been developed and tested in clinical trials. After a number of disappointing results, in 2022, a phase 3 randomized, controlled clinical trial showed, for the first time, that drug-induced reduction of amyloid beta plaque is accompanied by slowed decline on measures of cognition and function after 18 months of treatment (van Dyck et al., 2023). These data refer to the monoclonal antibody lecanemab that targets Aβ soluble protofibrils, approved by FDA early in 2023 (Reardon, 2023). Further data, supporting the clinical benefit of decreasing the Aβ burden in the AD brain are provided by the results of the phase 2 trial of the monoclonal antibody donanemab, targeting a pyroglutamate form of Aβ that is aggregated in amyloid plaques (Mintun et al., 2021; Gueorguieva et al., 2023 **)**. Top-line results of the phase 3 trial TRAILBLAZER-ALZ 2 (NCT04437511), confirming donanemab efficacy in slowing cognitive and functional decline in patients with early symptomatic AD, were recently released by Lilly (Donanemab 2023). The hypothesis of a causal role of Aβ in the pathogenesis of sporadic AD appears to be more soundly grounded now than it was in the past. However, the magnitude of the clinical effect elicited by both lecanemab and donanemab is limited, suggesting that additional pathological mechanisms may contribute to the disease.

Cumulative studies have recently paved the way to the recognition of neuroinflammation as an important co-actor in AD pathology, playing synergically with Aβ and p-tau cascades (Cribbs et al., 2012; Gomez-Nicola and Boche, 2015; Kinney et al., 2018; Knezevic and Mizrahi, 2018; Thakur et al., 2023). Consequently, several clinical trials focusing on neuroinflammation have been initiated and most of them have already entered phase 2 of development. In the present work, we briefly summarise the most recent data on neuroinflammation in AD and review the investigational drugs currently in clinical trials. We discuss their potential benefit/limitation based on their mechanism of action and their positioning in the pathologic cascade of events that occur in the brain throughout the course of AD. In addition, the latest patent requests for inflammation-targeting therapeutics to be developed in AD will also be presented.

## 2 The contribution of neuroinflammation in Alzheimer’s disease

The hypothesis of neuroinflammation as one of the key factors contributing to the pathogenesis of AD stood out from the widely accepted concept of inflammation as a by-product of pathological processes accumulating in the AD brain (i.e., Aβ plaques and p-tau-neurofibrillary tangles), when data from genome-wide association studies (GWASs) of sporadic AD cases showed associations between AD and genes involved in innate immunity (Kinney et al., 2018). This suggests that alterations in innate immune proteins, such as triggering receptor expressed by myeloid cells 2 (TREM2) and CD33, may increase the risk of developing AD (Naj et al., 2011; Bradshaw et al., 2013; Guerreiro et al., 2013; Jonsson et al., 2013). Neuroinflammation in AD is thought to be driven mainly by CNS-resident microglia and astrocytes.

### 2.1 Microglia

Microglia are the resident immune cells in the brain. They are able to rapidly respond to multiple danger signals and play important roles in tissue inflammation and clearance of cellular debris. In early AD, probably during the prodromal stage of disease (see the review by Cuello, 2017 for further insights), the activation of microglia resulted in the clearing of hyper-produced Aβ and confered initial protection against the disease by the production and release of anti-inflammatory cytokines (Simard et al., 2006). However, after prolonged exposure to Aβ, the protective activity of microglia faded and their overactivation induced changes in their gene expression profile, resulting in the production of pro-inflammatory cytokines, oxidative stress, neuroinflammation, and amplification of neuronal damage associated with Aβ and tau pathologies (Wolf et al., 2017; Hansen et al., 2018; Merlo et al., 2020; Zhang G. et al., 2021). High resolution transcriptome profiling has recently shown a complex microglial activation signature, encompassing four different microglia phenotypes: disease-associated microglia (DAM), which are characterized by downregulation of homeostatic genes, and upregulation of genes involved in inflammation, phagocytosis, cell survival, lysosome function, and lipid metabolism; two reactive microglia phenotypes in late response to neurodegeneration, typified by high expression of IFN-I-response (IFN-R) genes, and MHC class II (MHC II) genes; a forth microglial signature expressing markers of proliferation (Cycling microglia) (for a review see Hou et al., 2022). Trajectory analyses in the 5xFAD mouse (an AD-like mouse model expressing human mutant variants of the Aβ precursor, APP, and one of the APP protease, presenilin 1) showed that microglia progressively transit from a homeostatic state into the four distinct sub-populations (Ellwanger et al., 2021). How these different microglia phenotypes are produced and maintained during the course of the AD pathology, and which is their relevance and impact in the human disease is at present not known.

Microglia exposure to Aβ favoured the activation of NF-κB, MAP-kinase and NLRP3 inflammasome signaling cascades, resulting in the activation of caspase-1 and the secretion of pro-inflammatory cytokines IL-1β, IL-18 and TNF-α (Kelley et al., 2019). The activation of NLRP3 inflammasome is also involved in tau pathology. Loss of NLRP3 inflammasome function reduced tau hyperphosphorylation and aggregation; tau has been shown to activate the NLRP3 inflammasome, and intracerebral injection of fibrillar amyloid-beta-containing brain homogenates induced tau pathology in an NLRP3-dependent manner (Ising et al., 2019).

The ongoing inflammation may favour the formation of truncated and phosphorylated tau. As neurons can actively release non-fibrillar tau (Yamada et al., 2014), extracellular tau could contribute to chronic microglial activation and boost neuroinflammation (Khandelwal et al., 2012; Nilson et al., 2017; Rajendran and Paolicelli, 2018). A recent theory that tries to explain the progression from neuroinflammation to neuronal damage, moves from the observation of Aβ plaques even in post-mortem brains of subjects without AD dementia, and proposes that the speed of progression to AD dementia depends on the efficiency of microglia to clear the Aβ load and restrict the damage to the immediate vicinity of plaques. With the fading of the scavenger activity of microglia and the prevalence of their pro-inflammatory phenotype, the damage spreads along the axons, leading to the dissociation of tau protein from microtubules, axon and synaptic dysfunction and presentation of cognitive symptoms (Edwards, 2019). In line with this theory are the results of a recent PET imaging study in 130 AD patients showing that the interaction between Aβ and activated microglia determines how fast tau spreads across Braak stages (Pascoal et al., 2021).

The maintenance of the microglia pro-inflammatory phenotype could be also be favoured by the observed failure in the suppressive activity of regulatory T cells (T-regs) isolated from patients at the clinical Alzheimer dementia stage, but not at the MCI stage (Faridar et al., 2020). T-regs are a subset of T lymphocytes that maintain immune balance in the periphery, but also play anti-inflammatory and neurotrophic functions in the central nervous system (He and Balling, 2013; Liesz et al., 2013; Huang Y. et al., 2020). In particular, T-regs have been shown to suppress microglia-mediated inflammation by driving the differentiation of microglia towards a neuroprotective phenotype (Reynolds et al., 2009; Romano et al., 2018). The loss of T-reg suppressive activity in AD could thus contribute to maintain the immune system response in a pro-inflammatory phase.

Some of the most compelling data supporting a critical role of neuroinflammation in AD pathogenesis come from the demonstration that loss-of-function variants in the protein TREM2 confer up to a 4.5 fold increased risk of developing late onset AD (Guerreiro et al., 2013; Jonsson et al., 2013), positioning reduced TREM2 activity as the second strongest known risk factor for AD after *APOE* ε4 (Liu et al., 2013). TREM2 is a transmembrane protein receptor that is highly and exclusively expressed by microglia, and is both activated by Aβ as well as involved in Aβ phagocytosis (Parhizkar et al., 2019; Ellwanger et al., 2021). TREM2 signaling is required for the sequential activation of microglia from a homeostatic to a disease-associated state in response to Aβ exposure (Keren-Shaul et al., 2017). Impaired TREM2 function in the 5XFAD mouse reduced the proliferation and accumulation of microglia around Aβ plaques to limit their pathogenic potential (Wang Y. et al., 2015). This defective microglial response resulted in larger neuritic dystrophy and synaptic disconnection adjacent to Aβ plaque in AD early stages (Wang et al., 2016; Ulland et al., 2017). In humans, activation of TREM2, detected by increased cerebrospinal fluid (CSF) levels of soluble TREM fragment (sTREM), correlated with reduced Aβ and tau levels measured by positron emission tomography (PET) (Ewers et al., 2020). TREM2 also influences AD through modulating the inflammatory cascade. In microglia, the knockdown of TREM2 signaling increased TNF-α, IL-1β, IL-6, and nitric oxide synthase-2 transcription, whereas overexpression of TREM2 decreased gene transcription of TNF-α, IL-1β, and NOS2 (Takahashi et al., 2005; Jiang et al., 2014; Zheng et al., 2016). Downregulation of TREM2 expression induced cognitive dysfunction and exacerbated neuroinflammatory responses through the toll-like receptor 4 (TLR4) mediated MAPK signaling pathway in the APP/PS1 mouse model of AD (Ruganzu et al., 2022), while activation of TREM2 switched off the inflammatory response by inhibiting NLRP3 inflammasome and inflammasome complex assembly, through activation of beta-catenin (Wang et al., 2022).

Despite the large amount of evidence implicating TREM2 in anti-inflammatory and protective functions, a few reports have suggested an opposite role, favouring the appearance of a pro-inflammatory microglial phenotype (Jay et al., 2015; Tanzi, 2015; Zhong et al., 2017; Udeochu et al., 2018). A possible mechanism proposed for TREM2-driven microglial detrimental activation involves the binding to ApoE to promote dysfunctional features (Krasemann et al., 2017). Others have ascribed the detrimental functions of TREM2 to sTREM2, which in early symptomatic phases was shown to be elevated in the CSF and plasma of AD subjects, in association with higher phospho-tau levels (Heslegrave et al., 2016; Piccio L et al., 2016). An important role could also be played by infiltrating macrophages at some point in the progression of disease, with different functions compared to microglial TREM2 (Jay et al., 2015; Tanzi, 2015). Notably, the age of AD animal models lacking TREM2 impacted the effects on Aβ deposition, which was lower in earlier stages but exacerbated at later stages (Jay et al., 2017). Recently, the role of microglial TREM2 has been addressed in consideration of the link between Aβ and tau pathology. In AD mice at an age with evident plaque deposition, TREM2 enhancement with an agonist antibody significantly exacerbated tau pathology after brain injection of human-derived aggregated tau, without effects on plaque burden (Jain et al., 2023). These results are in contrast with previous studies from the same group, linking TREM2-deficiency to exacerbation of tau pathology in animal models (Leyns et al., 2017; Leyns et al., 2019). While opposing evidence for the role of TREM-2 in AD still need to be fully reconciled, context-dependent variations in TREM2 effects may have significant clinical implications for potential AD treatments that selectively target TREM-2.

The accumulating evidence of additional AD-associated gene variants expressed in myeloid cells further strengthens the role of microglia dysfunction and neuroinflammation in AD. In particular, the CD33 gene, expressed by myeloid cells, encodes a cell-surface protein that plays a critical role in inflammation by acting as a repressor of monocyte activation, upon regulation by sialic acid (Lajaunias et al., 2005). Loss-of-function of the CD33 gene was associated with reduction of insoluble Aβ levels in the AD brain, and increased levels of CD33 protein were observed in post-mortem samples of brain from AD patients (Bradshaw et al., 2013).

Microglia dysfunction induced by over-exposure to Aβ and/or mutations in microglia immune activity may thus establish a pro-inflammatory state that contributes to AD pathology.

### 2.2 Astrocytes

Astrocytes are specialized glial cells that, like microglia, are activated by all forms of CNS insults through a process referred to as reactive astrogliosis. Activated astrocytes provide neuroprotection by the release of neurotrophic factors; however, they may also favour neuroinflammation through the release of inflammatory cytokines and chemokines (Stadelmann et al., 2002; Farina et al., 2007). Astrocytes are able to clear Aβ (Lv et al., 2014), which in turn may activate astrocytes (Carrero et al., 2012). Reactive astrocytes surrounding senile plaques are a characteristic feature of AD (Wisniewski and Wegiel, 1991). Aβ deposits in astrocytes can be degraded by the action of metalloproteases, however Aβ-burdened astrocytes can undergo lysis to form astrocyte-derived amyloid plaques (Nagele et al., 2004). When astrocytes are chronically exposed to elevated Aβ levels, their neuro-protective potential decreases, their release of inflammatory mediators increases, (Ju Hwang et al., 2019), and their ability to supply reduced GSH to neurons and microglia fades, thus aggravating the pro-inflammatory state in the AD brain (Lee et al., 2010). In addition, astrocytes may contribute to Aβ-induced blood-brain barrier (BBB) damage through activation of endothelial metallo-protease, MMP9 (Spampinato et al., 2017). Indeed, the leakage of the BBB has been often observed in patients with early AD (van de Haar et al., 2016), and cerebrovascular lesions were seen, post-mortem, in AD patients (Zlokovic, 2011). Alteration of BBB may further worsen neuronal damage by decreasing Aβ clearance through the blood-brain barrier and depriving neurons from metabolic supply (Di Marco et al., 2015).

## 3 Materials and methods

### 3.1 Information sources and search strategies

To identify drugs targeting neuroinflammation in AD in preclinical development and in the development pipeline we used the US National Library of Medicine database of clinical trials at ClinicalTrials.gov^1^ and the World Intellectual Property Organization (WIPO) patient database^2^.

To retrieve relevant clinical trials from clinicaltrials.gov the following search term and criteria were used: Condition or disease “Alzheimer Disease”; Recruitment status “Recruiting, Not yet recruiting, Active, not recruiting, Completed, Unknown, Enrolling by invitation”; Study type “Interventional Studies.” The search cut-off date was 24/04/2023. The results were filtered to exclude trials describing behavioural therapy, devices, diagnostic tests, dietary supplements, procedures and other non-relevant interventions (e.g., music therapy, acupuncture, exercise, light therapy, virtual reality, *etc.*). Trials describing a pharmaceutical intervention were further reviewed to include agents targeting neuroinflammation. The following information was captured from the clinical trial record: the drug, sponsor, phase, status, start date, estimated end date, design of the trial, population enrolled, outcome measures (both primary and secondary endpoint). In addition, for each pharmaceutical agent the mechanism of action was described.

To retrieve patents describing potential pharmaceuticals targeting neuroinflammation in Alzheimer disease the WIPO Intellectual Property Portal was searched. The key word phrase “Neuroinflammation in Alzheimer disease” was applied in front page field which applies the entered value against the Title, Abstract, Numbers and Names. Results from all patent offices published in English up to a cut-off date 24/04/2023 were included. Patent results were reviewed and patents describing potential pharmaceuticals were included while other patents (e.g., those describing methods, assays, and apparatus) and duplicates were excluded. The patent details, drug, mechanism of action, stage of pre-/clinical development and background was captured from patent documentation and published literature.

## 4 Results

### 4.1 Clinical trials

A total of 2,226 clinical trial records were retrieved. 1,022 trials were excluded; 416 trials described behavioural therapy, 222 trials described a device, 24 trails described a diagnostic test, 55 trails described a dietary supplement, 55 trials described a procedure, and 250 trials described other non-pharmacological relevant interventions. The remaining 1,204 trials were reviewed, and 1,174 trial were further excluded as these trials described drugs that did not have neuroinflammation as their main target while 30 trials were included. These 30 trials described 18 investigational drugs of which 10 were small molecules, 7 were biologicals, and 1 was advanced therapy (Supplementary Table S1 in Data Sheet 1).

The majority of agents targeted intracellular inflammatory kinase signaling (4: NE3107, MW150, Neflamapimod a.k.a VX-745, baricitinib) or inhibited of the action or production of pro-inflammatory cytokines or eicosanoids (7: XPro1595 a.k.a Pegipanermin, Canakinumab, Lenalidomide, Emtricitabine, Montelukast, Salsalate, ALZT-OP1). Other agents modulated microglia and astrocyte activation (6: AL002, TB006, Edicotinib, Sargramostim, Pepinemab, Daratumumab). An agent exerted a broad-ranging immunomodulatory effect (1: VT301 a.k.a GB301).

### 4.2 Patents

20 patent entities were retrieved from the WIPO search. Seven entries were excluded. These included 4 patents which described a testing method [IN7506/DELNP/2013A/IN7506/DELNP/2013–CYREX LABORATORIES, LLC and WO/2007/008690/EP1915613 - Philadelphia Health and Education Corporation] and 3 patents that were duplicates [MXPA/A/2004/007292, WO/2003/064403 and AU2003207750 were duplicates of EP1478634 - Galileo pharmaceuticals inc.]. Supplementary Table S2 in Data Sheet 1 summaries 13 pharmaceutical agents or combinations patented for neuroinflammation in AD.

## 5 Discussion

The 18 investigational drugs targeting inflammation, currently under clinical investigation in AD, underlie three main clinical approaches to AD treatment: i. to promote a broad-ranging immunomodulatory effect; ii. to focus on the inflammatory signaling cascade generated through the course of the AD; iii. to target the CNS resident cells involved in neuroinflammation. While all three approaches have pros and cons, the one targeting CNS-resident microglia and astrocytes, that are considered the main drivers of neuroinflammation in AD, has recently received large attention in the scientific debate, and may be potentially more rewarding.

We will thus review all 18 individual agents but will provide a more detailed discussion on the group of investigational drugs regulating microglia and astrocytes activity (summary in Supplementary Table S1 in Data Sheet 1).

### 5.1 Broad-ranging immunomodulators

#### 5.1.1 VT301 (autologous regulatory T cells)

Regulatory T cells are thought to have a role in AD, although the relevance of their contribution and precise time frame of their involvement in the course of the pathology is at present a matter of debate. In a confocal microscopy study, significantly increased numbers of CD3^+^ extravascular T cells were observed in the brain of post-mortem AD patients, mostly in the hippocampus, compared to non-demented controls. The increase in CD3^+^ T cells correlated with tau pathology but not with amyloid plaques (Merlini et al., 2018), suggesting that T cell extravasation is driven by tau-related neurodegenerative changes and occurs in advanced stages of AD. A late involvement of T cells in AD is suggested also by the observation that T-reg suppressive activity towards inflammation decreased in patients at the clinical Alzheimer dementia stage, but not at the MCI stage, and that *ex vivo* expansion of T-regs from patients with AD restored T-reg activity (Faridar et al., 2020). Two trials using T-regs isolated from AD patients’ blood are listed in the clinicaltrails.gov website: the phase I/II trial NCT03865017 and the phase I trial, NCT05016427. They are both run by VT BIO a biotechnology company that develops cell therapies for neurodegenerative diseases, headquartered in Seoul. The phase I trial, after a substantial delay, was started at Seoul National University Hospital. The Company announced on 6 July 2022 (VTbio Heath Korea News) that the domestic phase 1 clinical trial was scheduled to be completed in the second half of 2022, and based on the results, the second trial was planned to start the first half of 2023; the latter was originally planned to start on December 2019. On 23 June 2022, VT BIO said the company received approval to conduct clinical trials of its VT301 drug by the FDA (koreabiomed published information). No additional information is currently available.

### 5.2 Drugs interfering with the inflammatory signaling cascade: kinase inhibitors

#### 5.2.1 P38 MAPK inhibitors

Two investigational drugs inhibiting p38 MAPKs are currently in clinical trials: Neflamapimod (NCT03402659) and MW150 (NCT05194163). P38 MAPK are activated by various cell stressful or noxious stimuli and mediate the inflammatory response (Cuenda and Rousseau, 2007). Of the 4 p38MAPKs expressed by mammals (D'Mello, 2021), p38α and p38β are the most studied. Both of them are expressed in neurons as well as astrocytes, microglia and oligodendrocytes (D'Mello, 2021).

In microglia and astrocytes, p38MAPKs activation by APP and Aβ resulted in the release of inflammatory cytokines (McDonald et al., 1998; Giovannini et al., 2002; Kheiri et al., 2018). In neurons, p38α MAPK increased pathogenic p-tau and promotes neuronal damage (Goedert et al., 1997; Roy et al., 2015; Maphis et al., 2016). In mouse AD models, inhibition of p38α was protective against inflammation and synaptic dysfunction and ameliorated cognitive functions (Munoz et al., 2007). In addition, p38α was reported to inhibit autophagy (He et al., 2018). In the human AD brain, the expression of activated p38MAPKs seems to increase transiently, with higher levels detected in neurons at the early stage of tau pathology, but not in typically NFTs (Sun et al., 2003). Several selective inhibitors of p38 MAPK have been synthesized (Dominguez et al., 2005), of which neflamapimod (VX-745) and MW150 are able to penetrant the brain (Roy et al., 2019; Tormählen et al., 2022).

##### 5.2.1.1 Neflamapimod

Neflamapimod, previous referred to as VX-745, was initially tested for rheumatoid arthritis, but was later discontinued, due at least in part to the occurrence of CNS toxicity, in preclinical studies (Haddad, 2001). Interest in this agent was later renewed due to its favourable brain permeability index, with drug twice higher concentrations in the CNS than peripheral blood (Tormählen et al., 2022)**.** A first phase 2a, exploratory trial in patients with MCI or mild AD (MMSE 20–28, biomarker positive) showed that a 6-to-12-week of neflamapimod treatment partially reduced in amyloid PET (only one of the two primary endpoints on amyloid burden was met), and caused a statistical significant improvement in episodic memory, measured as secondary endpoint (Scheltens et al., 2018). However these results were not confirmed in a phase 2b 6-month placebo-controlled trial (NCT03402659) in 150 MCI or mild AD patients treated with a 6-month course of a 40 mg neflamapimod taken twice daily. No difference from placebo was observed either in the primary endpoint, total and delayed recall on the Hopkins Verbal Learning Test Revised (HVLT-R), nor in secondary endpoints including Wechsler Memory Scale (WMS) Immediate and Delayed Recall, CDR-Sum of Boxes, and MMSE. In pre-specified subgroup analyses, patients with the highest plasma drug concentrations showed a positive trend towards improvement relative to placebo on both HVLT and WMS. Neflamapimod treatment also induced statistically significant reductions in the biomarker, CSF phospho-tau, and a trend toward reduced neurogranin (Prins et al., 2021). In their comment to the published results (May 2021), the study investigators anticipated a future study of longer duration and higher dose of neflamapimod to assess the effects of the drug on AD progression. However, at present, such study has not been started, and the only other study listed on clinicaltrials.gov website is a proof-of-concept study to measure the evolution of neuroinflammation in the brain of MCI or mild AD patients, after 12 weeks of treatment. This study was expected to run until June 2021, and no update is currently available.

##### 5.2.1.2 MW150

MW150 is a kinase inhibitor fragment selective for the p38αMAPK. Pharmacokinetic assays demonstrated that MW150 has good oral bioavailability, high cell permeability, and favourable distribution across the blood-brain barrier (Roy et al., 2015; Roy et al., 2019). According to the information published on the Company website (NeuroKine Therapeutics), the drug proved safety and well-tolerated in a phase I trial in healthy individuals. In January 2022, a phase II trial (NCT05194163) was listed on the clinicaltrials.gov website evaluating MW150 in patients with mild to moderate AD who will receive 10 mg daily capsule of the drug for 12 weeks. Currently the trial is not yet recruiting. The primary outcomes is safety. Secondary outcomes include measures of cognition, daily function, neuropsychiatric symptoms, and blood levels of cytokines, tau, and neurofilament light. The trial is expected to run until August 2024.

#### 5.2.2 The ERK/NF-kB inhibitor NE3107

NE3107 is an insulin-sensitizing, orally bioavailable small molecule that binds to ERK and reduces inflammation-driven ERK- and NF-κB-stimulated inflammatory mediators, without interfering with their homeostatic functions (Reading et al., 2021). Activation of ERKs has recently stand out as an important inflammatory kinase signaling pathway in microglia from AD transgenic mice, and in post-mortem brain from AD subjects (Chen et al., 2021). NF-κB is predominately found in neurons and glial cells that surround Aβ plaques and its activation, which is triggered by Aβ and P-tau, modulates the production of pro-inflammatory cytokines and plays a central role in reactive microglia (Kinney et al., 2018).

NE3107 has completed an open-label, single arm phase 2 trial in 23 patients, 18 patients with MCI or mild AD, and 5 patients with MMSE <20 (i.e., moderate AD) (NCT05227820). Primary endpoints were AD-related brain changes measured with functional MRI, in patients treated with 20 mg of NE3107 twice daily for 3 months. Secondary outcomes included changes in cognition through verbal and visual test procedures, and changes in CSF AD markers. Top line results were released on 7 September 2022 (NCT05227820 results, 2022), and additional data were presented at the Clinical Trial in Alzheimer’s Disease annual conference, held in San Francisco, in 2022 (Rindner et al., 2022). According to the Investigators, the majority (18) of 22 patients with abnormal baseline scans showed improvement in one or more brain regions as seen with advanced fMRI. Patients also experienced a reduction in CSF p-tau levels of −1.66 pg/mL (*p* = 0.0343) and in the ratio of p-tau to Aβ42 of −0.0024 (*p* = 0.0401). The majority (62%) of 13 MCI/mild AD patients had decreased plasma TNF levels with a mean change of −0.55 pg/mL. Encouraging results were also observed in clinical endpoints with 82% of 17 patients experiencing a 2.6-point decrease in ADAS-Cog12. In January 2022, the Company started a phase III trial to evaluate NE3107 in patients with mild to moderate Alzheimer’s disease (NCT04669028). The study has co-primary endpoints looking both at cognition, using the ADAS-Cog 12 scale, as well as function, using the ADCS-CGIC scale. Top line results are foreseen in October 2023.

#### 5.2.3 The Janus kinase inhibitor baricitinib

Baricitinib is approved for treatment of rheumatoid arthritis by both FDA and EMA, and, limited to Europe, for atopic dermatitis. The JAK/STAT pathway is the predominant signaling pathway used by cytokines and is central for both innate and adaptive immunity. Activation of JAK/STAT3 has been observed in astrocytes in many conditions and disease models, including AD (Villarino et al., 2017). In microglia isolated from the brain of the APP/PS1 mice, pharmacological inhibition of JAK2 attenuated IFN-γ-induced expressions of pro-inflammatory cytokines (Jones et al., 2015). In contrast to the above findings, decreased levels of phospho-STAT3 were reported in hippocampal neurons of AD patients and in a mouse model of AD, and inhibition of the JAK2/STAT3 pathway resulted in spatial working memory impairment and cholinergic dysfunction (Chiba et al., 2009). These data suggest that inhibitors of the JAK2/STAT3 pathway may have context-dependent effects, and that their use as therapeutics in AD may be complex. However, a machine learning study of gene expression profiles of AD brains identified Baricitinib as one of the kinase inhibitors that reversed the impaired inflammatory signaling in AD (Rodriguez et al., 2021). The drug is thus considered potentially promising for repurposing in AD. Baricitinib is being currently evaluated in a phase I/II basket trial in 20 patients with MCI, mild AD or Amyotrophic Lateral Sclerosis who must have elevated levels of the inflammatory cytokine CCL2 in CSF (NCT05189106). Participants will be treated for 24 weeks with the primary aim to evaluate the brain permeability and anti-inflammatory activity of the drug by measuring CSF concentration of both Baricitinib and CCL2. Safety will be assessed as secondary endpoint together with a number of inflammatory molecules and AD biomarkers. Indeed, the main limit to the potential use of Baricitinib in AD is its problematic safety profile, including increased risk of serious infections and cancer, major cardiovascular events, blood clots, and death.

### 5.3 Inhibitors of the action of pro-inflammatory cytokines

#### 5.3.1 TNF inhibitor XPro1595

TNF-α is a central actor in neuroinflammation. Its levels are significantly elevated in blood (Fillit et al., 1991) and CNS (Tarkowski et al., 2003) of patients with AD. In animal models of AD, TNFα favoured microglial activation and accumulation of β-amyloid plaques, synaptic dysfunction, and cognitive decline (Chang et al., 2017). TNF-α binds to 2 receptor subtypes with different signaling cascades, TNFR1 and TNFR2 which are activated by both soluble and transmembrane forms of TNF or mainly by transmembrane TNF, respectively. Although the effects of TNF receptor activation are multiple and context-dependent, TNFR1 activation is characterised by pro-apoptotic activity, whereas TNFR2 typically promotes cell survival, proliferation and maintenance of innate immune function (McCoy and Tansey, 2008). Several non-selective TNF-α biologic inhibitors are approved for use in the treatment of peripheral autoimmune disorders and are thus potential candidate as AD therapeutics; however, their poor brain penetration limits their use in the clinical setting. The monoclonal antibody Infliximab was shown to decrease p-tau, and Aβ plaque burden when injected intracerebroventricular in a mouse model of AD (Shi et al., 2011). Encouraging results on cognitive performances were obtained, in an open-label study, with the fusion protein Etanercept administered via perispinal injection, in 15 patients with mild to severe AD, for 6 months (Tobinick et al., 2006). However, these data were not confirmed when etanercept was administered s.c. to 20 patients over 24 weeks in a double-blind placebo-controlled study (NCT01068353; Butchart et al., 2015). Given the etanercept limited brain permeability, the lack of efficacy reported in this study indicates that the strategy to target peripheral systemic inflammation occurring in AD patients, with anti-TNF drugs, may be not productive.

The second-generation, non-receptor binding variant of TNFα, XPro1595, is brain permeable and forms heterotrimers with native soluble TNFα, preventing its activation of TNFR1 (Steed et al., 2003). XPro1595 does not block TNFR2-mediated signaling and thus does not negatively impact on innate immunity or myelination (Zalevsky et al., 2007; Brambilla et al., 2011). The selective inactivation of TNFR1 receptor by XPro1595 should thus result in a better safety profile, avoiding potential neurological problems and limiting drug-induced increased susceptibility to infections and cancer that are characteristics side effects of the non-selective TNF inhibitors. Of note, long-term treatment of healthy adult mice with etanercept, but not with XPro1595, decreased neurogenesis in the hippocampus and impaired spatial learning and memory (Yli-Karjanmaa et al., 2019). Four clinical trials are currently listed on clinicaltrials.gov website. The phase Ib open-label study NCT03943264 evaluated s.c. XPro1595 treatment once a week for 3 months in 16 mild to moderate-severe AD patients with signs of inflammation. The primary endpoint was safety, while secondary endpoints included changes in blood/CSF inflammatory and AD biomarkers, MRI measurements of brain oedema, axonal degeneration and demyelination, and cognitive aspects. After completion of the 3-month treatment, patients could enter an extension study up to a total 1 year of treatment. Study results were not published but are available on videos on the Company website (INmune Bio, 2021). According to the Company, XPro1595 showed an acceptable safety profile, with injection site reactions being the main adverse event. Brain neuroinflammation, as shown by white-matter free water, was decreased by 5% after 3 months and, in the 3 patients that completed the extension study and were treated with the highest dose, by 46% after 12 months. Encouraging results were also observed in fibre density, a marker of axonal integrity, and remyelination. Participants also showed decreases in multiple inflammatory proteins and p-tau in the CSF. These results prompted the planning of two phase II studies investigating XPro1595 in mild AD patients (NCT05318976) and in MCI patients (NCT05321498), respectively, and a forth open-label follow-up study that will enroll patients from the two phase II trials. Both phase II studies have the same primary outcome, 6-month changes in the Early and Mild Alzheimer’s Cognitive Composite; the trial in mild AD patients is currently recruiting, while the one in MCI patients, although scheduled to start in May 2022, is still not recruiting.

#### 5.3.2 IL-1 inhibitor, canakinumab

Canakinumab is an IL-1β neutralizing antibody approved in various inflammatory diseases. Increased serum levels of IL-1β have been associated with AD (Forlenza et al., 2009) and IL-1β polymorphism correlated with age at onset of AD (Sciacca et al., 2003; Payão et al., 2012). In the AD brain, the activation of NLRP3 inflammasome signaling cascade in microglia led to caspase-1 activation and consequent proteolytic cleavage of pro-IL-1β in active IL-1β (Kelley et al., 2019). Biological agents targeting IL-1β mainly include IL1β antibody canakinumab and recombinant IL-1β receptor antagonist anakinra, which share some approved indications. In animal models of AD and in mice injected with Aβ oligomers, anakinra was reported to reduce brain inflammation, p-tau, cognitive deficits, synaptic loss and ameliorate cognitive impairment (Kitazawa et al., 2011; Batista et al., 2021). However, the non-negligible safety profile of the currently available anti-IL1β drugs, their brain limited penetration and the multiple inflammatory signals downstream to NLRP3 inflammasome activation may limit their repurposing in the treatment of AD (Liang et al., 2022). The potential for canakinumab as a therapeutic in AD, is currently investigated in one phase II trial in patients with MCI or mild AD treated for 20 weeks and followed up for additional 28 days. The primary endpoint is change from baseline in cognition as measured by the Neuropsychological Test Battery (NTB) total score. Among secondary endpoints, the trial includes safety, changes in microglia activation, functioning and neuropsychiatry measurements. The trial was due to end in 2024 but has been delayed to February 2026 (NCT04795466).

### 5.4 Inhibitors of the production of pro-inflammatory cytokines or eicosanoids

#### 5.4.1 Inhibitors of pro-inflammatory cytokine production, lenalidomide and emtricitabine

##### 5.4.1.1 Lenalidomide

Lenalidomide is a widely used immunomodulatory drug with multiple mechanisms of action, including a potent inhibition of TNF-α and other inflammatory cytokines (Muller et al., 1999). Several studies have tested the efficacy and safety of lenalidomide outside the marketed indications, in clinical conditions in which inflammation is central to the pathology, showing amelioration of persistent central inflammatory damage (Liu X. et al., 2022). Lenalidomide is currently under investigation in a Phase II, double-blind, randomized, placebo controlled study in 30 amnestic MCI patients. Treatment effect will be assessed after 12 months of treatment and 6 months washout. Primary endpoint of the study will be the evaluation of change in cognition and functioning by a battery of neuropsychological scales. Platelet and neutrophil toxicity, and amyloid burden, CNS neurodegeneration and blood inflammatory markers will be secondary endpoints (NCT04032626).

##### 5.4.1.2 Emtricitabine

Emtricitabine belongs to the class of nucleoside reverse transcriptase inhibitors (NRTIs) used in the treatment of HIV, which has been shown to reduce neuroinflammation by inhibiting the activation of the NLRP3 inflammasome (Fowler et al., 2014) and/or alleviating the effects of retrotransposon activation upstream to IFN signaling cascade (De Cecco et al., 2019). It is currently being evaluated in a phase 1 trial (NCT04500847) aimed at characterizing the drug tolerability in MCI and mild to moderate AD patients. In fact, although emtricitabine shows a more manageable safety profile compared to other NRTI drugs in HIV patients, data on its use for long-term treatment in the elderly population are limited and further characterization is needed.

#### 5.4.2 Inhibitors of the production of eicosanoids

Although epidemiological studies seemed to indicate that people treated chronically with non-steroidal anti-inflammatory drugs have a decreased risk of AD (Stewart et al., 1997), the large majority of clinical trials have failed in proving the efficacy of NSAIDs in symptomatic AD patients. Several reasons could account for the observed lack of efficacy: inappropriate timing of the treatment which was given mostly in advanced AD patients, suboptimal drug brain permeability, or the fact that prostanoids are only minor players in the AD pathology.

Although not considered at present among the most promising therapeutic strategies for AD, a number of eicosanoid inhibitors are currently under clinical investigation. In all cases, additional mechanisms of action have been proposed for their potential use in AD.

##### 5.4.2.1 Salsalate

Salsalate is NSAID selected as a drug candidate for AD due to its ability to inhibit p300 acetyltransferase and by that reduce tau acetylation (Min et al., 2015). Acetylation at Lys174 is a toxic alteration of the soluble tau protein that occurs early in the AD pathology and promotes tau aggregation, neuronal damage and cognitive deficits (Min et al., 2010; Cohen et al., 2011). Encouraging results in a mouse model of tauopathy, in which salsalate decreased acetylation of tau protein, rescued tau turnover and ameliorated cognitive performance (Min et al., 2015), led to the initiation of a phase 1b 12-month, randomized, double-blind, placebo-controlled study (NCT03277573), in patients with mild to moderate AD, investigating safety and tolerability as primary endpoint and, among others, measurements in blood and CSF salsalate and changes in CSF biomarkers and cognition as secondary and exploratory endpoints, respectively. The trial was completed in December 2021 and results were published in abstract form (Ljubenkov et al., 2022). Although salsalate treatment was safe and well tolerated (no information on gastric protection in the recruited patents is although available) no drug-induced positive trend in CSF biomarkers or clinical measures was observed. However, no tau PET analysis to verify target engagement is available. The Investigators mentioned a high trial dropout rate and baseline differences in cohorts as potential confounders.

##### 5.4.2.2 ALZT-OP1

ALZT-OP1 is a combination of two well-known marketed drugs: the NSAID ibuprofen and the mast cells stabilizer cromolyn, currently approved for asthma treatment. Cromolyn was reported to promote microglia phagocytosis of Aβ (Zhang et al., 2018), and both cromolyn and ibuprofen were shown to decrease Aβ aggregation in mouse models of AD, with increased efficacy observed with combination therapy (Lim et al., 2000; Zhang et al., 2018). Currently, two clinical studies are listed in clinicaltrials.gov: a phase I/II trial (NCT04428775) to investigate PK/PD and safety of ALZT-OP1 in both healthy subjects and mild to moderate AD patients, and a phase III, 18-month trial (NCT02547818), in 620 early AD patients, the primary outcome of which was change in cognition and function. The two trials were concluded in January 2021 and November 2020, respectively with no posted or published results. Of note, in the two trials, cromolyn was administered by inhalation, a root that optimises site-specific activity and confers a good safety and tolerability profile when the drug is given for the treatment of asthma. However, the limited systemic absorption obtained through this root may result in insufficient brain penetration and limited efficacy in the AD indication. Indeed, cromolyn absorption through the gastrointestinal tract is also very poor, being less than 1% of the administered dose. Results from the PK/PD and safety study are thus eagerly awaited.

##### 5.4.2.3 Montelukast

Montelukast is a cysteinyl leukotriene receptor antagonist approved for the treatment of asthma and allergy symptoms. Neurons and microglia express leukotriene receptors, and upregulation of CysLT_1_R was correlated with increased Aβ and APP, and associated with cognitive dysfunctions in mice (Wang X. Y. et al., 2013;; Tang et al., 2013). In preclinical studies, montelukast was reported to reduce neuronal damage and memory deficits in mice that received intracerebral Aβ infusions, (Lai et al., 2014), and decrease neuroinflammation, favour hippocampal neurogenesis and improve learning and memory in old animals after a 6-week oral treatment (Marschallinger et al., 2015). A phase II randomised, placebo-controlled trial using a modified formulation of montelukast, with enhanced bioavailability and BBB permeability (IntelGenx, 2019), is currently ongoing in 70 mild to moderate AD patients. The primary endpoint is changes in global neuropsychological test battery composite scale, and additional cognition and functional testing are included together with safety as secondary endpoints. The study was suspended in late 2020 due to the COVID-19 pandemic and resumed in January 2022 with end-of-study postponed to December 2023. An independent academic double blind 1-year phase II trial (NCT03991988), using unmodified montelukast tablets and initially intended to assess the safety of escalating dose of the drug in 150 MCI and mild AD patients, was completed in November 2022 with only 32 patients recruited. Results are still not available.

### 5.5 Agents modulating microglia and astrocyte activation

#### 5.5.1 AL002

AL002 is an anti-human TREM2 agonistic monoclonal antibody (mAb). The identification of TREM2 deficient genetic variants, as an important risk factor in AD, has made TREM2 a potential target for therapeutic strategies in AD. However, the selection of the patient population may be critical for the evaluation of the efficacy of the agonistic TREM2 mAb, as some studies have proposed that the effect of TREM2-mediated microglia activation varies with the disease stage. TREM2 deficiency was associated with reduced Aβ load at early stage of plaque formation (Jay et al., 2015), but exacerbated amyloid pathology late in disease progression (Jay et al., 2017). Overexpression of non-cleavable TREM2, in the APP23/PS45 mouse, resulted in sustained TREM2 stabilization and increased numbers of small plaques, but not medium and large plaques (Dhandapani et al., 2022). Opposite results were obtained with short-term administration of TREM2 knockdown antisense oligonucleotides to APP/PS1 mice at varying stages of plaque pathology. In the advanced stage, in 10-month-old mice, plaques were reduced by half, whereas no effect was observed when the TREM2 knockout was performed in early AD stages (Schoch et al., 2021). Overall data suggest a time- and/or dose-dependent role for TREM2 in mediating plaque deposition and microglial responses and that efficacy of TREM2-based therapeutic strategies may be restricted to a certain time window. Several monoclonal antibodies activating TREM2 have been developed (Cheng et al., 2018; Schlepckow et al., 2020; Ellwanger et al., 2021; Zhao P. et al., 2022), although only one is at present in the clinical phase. Preclinical data showed that administration of the hTREM2 agonistic mAb AL002c, in the 5XFAD mouse expressing the hTREM2 transgene, acutely expanded proliferating microglia and, following chronic exposure, reduced filamentous Aβ plaques and neurite damage (Wang S. et al., 2020). However, no reduction of total Aβ load nor increase in microglia clustering around Aβ plaques, which limits spreading and neuronal toxicity, was observed, differently from what expected based on data from a different TREM2 activating mAb (Schlepckow et al., 2020) and previous independent observations in 5XFAD mice (Wang Y. et al., 2015). No dedicated study on the potential effect of AL002 on memory and learning is at present available, although minor changes in risk-taking and anxiety-like traits were reported using the 5XFAD mouse. Of note, alarming results came from a recent study where the murine variant AL002a was chronically administered to 6 months-old 5xFAD mice to study the effect on tau pathology, after the ipsilateral injection of aggregated tau from human AD brains. Unexpectedly, while plaque burden was unaffected, tau pathology worsened along with loss of synaptic proteins and neuritic dystrophy (Jain et al., 2023). Enhancing TREM-2 activity in a stage with ongoing Aβ-driven dysfunctions may thus elicit a completely different outcome compared to TREM2 deficiency since birth, as in knock-out AD mice. These findings, raise an important issue regarding the long-term safety of AL002 in humans, highlighting once more the need to select the right population for a successful therapy. In the case of AL002 the extent of tau pathology is suggested to be a critical factor.

In the first-in-human phase I clinical trial of AL002, 56 healthy adult participants received a single i.v. dose of the drug and were followed out to 12 weeks after dosing (NCT03635047). According to the short information published in the paper by Wang Z. F. et al. (2020), the safety and tolerability profile was acceptable with no drug-related serious adverse events or dose-limiting toxicities up to the highest tested dose (Wang S. et al., 2020). A dose-dependent decrease in sTREM2, the product of proteolytic cleavage of the cell-surface TREM2, was detected in the CSF shortly after dosing, paralleled by an increase in sCSF-1R, the cleavage product of transmembrane CSF-1R, which is only expressed by microglia in the brain. TREM2 cleavage prevents TREM2 ability to transduce intracellular signals. However, sTREM2 can trigger an independent signaling pathway, by activating the PI3K/Akt cascade, and enhance microglial proliferation, migration, uptake and degradation of Aβ (Zhong et al., 2017; Zhong et al., 2019). In particular, data by Zhong et al. (2019) showed that administration or induced expression of sTREM2 in the 5xFAD mouse model of AD reduces amyloid plaque load, and favours recovery of spatial memory, in a microglia-dependent way Zhong et al., 2019). It will need to be assessed if changes in sTREM and sCSF-1R CSF levels are maintained during prolonged treatment in AD patients and play a role in the potential therapeutic effect of AL002.

A phase 2 RCT with AL002 was initiated in January 2022 aimed at enrolling 255 adults with AD. The trial population will be selected based on positive PET amyloid or CSF biomarkers, coupled with a score at the cognitive scale MMSE ≥22 points, a CDR-global score between 0.5 and 1, and a score on the delayed memory index of the RBANS scale ≤85. Altogether, these inclusion criteria should allow the recruitment of an enriched patient population with MCI due to AD or mild AD dementia who will receive i.v. AL002 every 4 weeks up to 48 weeks through 96 weeks. Hopefully, study results will give us further indications on the correct patient population to assess the efficacy of AL002 (NCT04592874). A long-term extension study to evaluate the safety, tolerability, and efficacy of AL002 in participants with early Alzheimer’s disease was initiated in February 2023 (NCT05744401).

An additional complexity in predicting the potential efficacy of TREM2 targeted therapies in AD derives from the heterogeneity of the microglia subsets responding to AD pathology. Limited information is at present available on the different microglia subtypes interplay and impact on the progression of the disease. The DAM population was shown to secrete CSF1 that may sustain survival and proliferation of microglia around plaques (Otero et al., 2009; Song and Colonna, 2018). In contrast the type I IFN population which is associated with larger neuroinflammation and cognitive decline in AD (Roy et al., 2020) may inhibit CSF1-stimulation and cell cycling (Hamilton, 1997). Therefore, different microglial subsets may have beneficial or detrimental impacts on AD pathology, and the overall impact of TREM2 targeting therapies may depend on the microglia status of the AD brain.

Another interesting molecule targeting TREM2 is an agonistic antibody engineered to be more easily delivered across the BBB thanks to a monovalent transferrin receptor binding site, acting as an antibody transport vehicle (ATV). The molecule has been designated as ATV:TREM2 or DNL919 (van Lengerich et al., 2023). ATV:TREM2 has not yet been tested in AD patients, but showed improved brain distribution and signaling compared to a standard anti-TREM2 antibody. In addition, microglial metabolic activity and glucose metabolism appeared elevated as by TSPO-PET and FDG-PET imaging, while shedding of sTREM2 was reduced. Improvement of metabolic and proliferative microglial features were confirmed *in vitro* on iPSC-derived microglia, and single-cell RNA sequencing interestingly showed that this state was different from the Aβ-induced activated state (van Lengerich et al., 2023). These results had been preceded by studies on a mouse version of the antibody, shown to effectively enhance plaque clearance in a mouse model of amyloidosis targeting microglia through TREM2 signaling (Schlepckow et al., 2020).

DNL919, entered a phase 1 clinical trial (NCT05450549) in Europe in July 2022, to investigate safety, tolerability, pharmacokinetics and target engagement after single ascending doses. The study is currently recruiting, with an estimated enrollment of 80 healthy participants, and results are expected by July 2023. In US, the clinical investigation of the drug was halted by FDA in January 2022, pending clarifications on preclinical toxicology, issues in the clinical trial protocol and additional administrative matters (Denali Therapeutics press release, 2022; SEC disclosure, 2022).

#### 5.5.2 TB006

TB006 is a humanized monoclonal antibody targeting galectin 3, a ubiquitously expressed β-galactosidase-binding lectin, able to interact with target partners to activate cell adhesion, migration, differentiation, as well as immune and neuroinflammatory responses (Hara et al., 2020; Mijailović et al., 2022). Recently, Galectin 3 has been implicated in the development of a number of diseases such as cancer, stroke, and inflammatory-driven pathological conditions (Dumic et al., 2006; Henderson and Sethi, 2009; Rahimian et al., 2021). In particular, data from single cell transcriptomic analyses have shown that upregulation of galectin-3 is a shared feature among different populations of specific neurodegenerative disease-associated microglia, including AD (Holtman et al., 2015; Butovsky and Weiner, 2018; García-Revilla et al., 2022; Mijailović et al., 2022). Pro-inflammatory activation of microglia has been ascribed to extracellular galectin-3 signaling, in particular to the interaction with the TLR-4, a key mediator of inflammation, as opposed to anti-inflammatory signaling mediated by the interleukin 4-receptor-PPAR-γ pathway (Mijailović et al., 2022). Moreover, microglia have been shown to secrete galectin-3 in response to LPS *in vivo*, giving life to a pro-inflammatory loop involving paracrine TLR4-mediated signaling (Burguillos et al., 2015).

Genomic association studies confirmed variants of galectin-3 as risk factors for AD (Trompet et al., 2012; Boza-Serrano et al., 2019), and galectin-3 levels in blood or CSF were shown to be elevated in AD patients (Wang X. et al., 2015; Yazar et al., 2021; Boza-Serrano et al., 2022). Galectin 3 was reported to promote Aβ oligomerization, associated with increased neuroinflammation and cognitive impairment; accordingly its knockdown produced opposite effects, compared to wild type animals, following hippocampal injection of Aβ (Tao et al., 2020). Moreover, galectin 3 was secreted by microglia and directly activated TREM2, which in turn promoted the upregulation and release of more galectin 3 *in vitro* (Tao et al., 2020). Finally, in the same study, Aβ oligomers and galectin 3 were increased in the frontal lobe of AD patients. These observations were confirmed by data showing upregulation of galectin 3 in microglia exposed to Aβ *in vitro*, in microglia surrounding amyloid plaques *in vivo* and in specimens from AD patients (Boza-Serrano et al., 2019). Genetic knock-down of galectin 3 in an AD mouse model significantly reduced pathology and improved cognition (Boza-Serrano et al., 2019).

Based on these observations, the development of a selective anti-galectin 3 antibody as an anti-AD drug is being pursued with high hopes. It is worth noting that, even though the rationale for use of TB006 in AD therapy stems from a classic amyloid-centric view of AD pathogenesis, the combination of an indirect approach to reduce Aβ build-up, upstream of its aggregation into toxic species, and a direct interference with microglial inflammatory activation looks innovative. These combined effects hold the potential to inhibit the vicious cycle between pro-inflammatory microglial activation and Aβ accumulation/aggregation early on in disease development, in line with the latest approaches aiming at shifting diagnosis and treatment to the earliest possible stages of disease.

Three clinical trials are currently under way for TB006 in AD. A phase 1 randomized, double-blind, single-dose, dose-escalation study healthy adults (NCT04920786) was started in June 2021 and is still recruiting. Results were so far positive, with good safety and tolerability profiles (Sun et al., 2022). The second study (NCT05074498) is a seamless phase 1b/2 trials aimed at further investigating the safety of TB006 while testing drug short-term efficacy, in mild to severe AD patients (MMSE score ≤24 and age ≥50). Aβ positivity was not taken into account as a requirement. In the phase 1b portion, three groups of eight patients received either weekly TB006 or placebo infusions in sequential ascending fashion for one month. In the phase 2 portion, participants were randomized (1:1) to receive either TB006 (at the highest dose,1,000 mg) or placebo weekly for one month. Primary endpoint was safety. Other endpoints were the MMSE, neuropsychiatric inventory, CDR batiery and plasma and imaging (MRI/PET) biomarkers. According to the published information on the Company website (TB006 phase1b/2), a trend towards amelioration of cognitive function not reaching statistical significance, was observed for the primary efficacy endpoint. TB006 differed from placebo by 63% (*p* = 0.08) on CDR-SB, after completion of the 104 days program. This coupled with a reduction of β-amyloid 42 CSF levels, but with no changes in the Aβ42/40 ratio, p-Tau181, or neurofilament light chain (NfL), while amyloid plaques were reported to be reduced, as detected by PET scan. These data are only preliminary and will need to be further confirmed but appear especially relevant given the short time of treatment needed to achieve initial cognitive benefits.

The study was extended, starting July 2022, as an open-label, long-term trial (NCT05476783) enrolling patients who completed the lead-in study in addition to *de novo* patients with the same eligibility criteria, for a total of 180 participants enrolled. The drug is administered monthly at a higher dose over 101 weeks with a 12-week safety follow-up period. According to the Company’s press release, in late Aprile 2023, interim data showed that among the 79 participants who completed a 3-month TB006 regimen, 47% had signs of disease reversal or cognitive improvement, and 28% had stabilized disease.

#### 5.5.3 Edicotinib

Edicotinib, also termed JNJ-40346527, is an oral, selective inhibitor of the colony-stimulating factor-1 receptor tyrosine kinase (CSF1R). In N13 microglial cells, edicotinib led to a dose-dependent decrease of CSF1R activation and a concurrent reduction of ERK1 and ERK2 phosphorylation. Microglial viability and proliferation depend on signaling through the CSF1R (Waisman et al., 2015), the expression of which is significantly increased in neurodegenerative diseases. Administration of CSF1R antagonists in mice induced rapid apoptotic microglial death, followed, upon discontinuation of drug treatment, by a robust proliferation of the residual microglia, leading to repopulation, phenotype changes and normal cellular density (Elmore et al., 2015; Olmos-Alonso et al., 2016). Microglia depletion following CSF1R inhibitors may be limited to specific cellular subsets, as reported by Spangenberg et al. (2019), who showed a selective depletion of plaque-associated microglia in their AD model. Importantly, both the CSF1R inhibitors-resilient microglia and the newly repopulated microglia show a more homeostatic phenotype that confers them a remarkable brain repair activity (Elmore et al., 2018; Han et al., 2019). Based on these data, it has been speculated that the renewed microglia, could more effectively afford protection against AD by better compacting amyloid plaque and preventing diffusion of damage. However, conflicting results were generated when this hypothesis was tested preclinically. While changes in the distribution of Aβ were observed when CSF1R inhibitors were administered at the beginning of the pathology, the majority of reports showed that microglial depletion in Aβ mouse models resulted in no changes in total Aβ burden. In one report, CSF1R inhibitors increased the number of dystrophic neurites (Casali et al., 2020), while in other studies they reduced neuritic plaques and cognitive decline (Sosna et al., 2018). Given the heterogeneity of microglia phenotypes in AD, these contrasting results are not surprising as may depend on the particular microglia subtype combination background in which the CSF1R inhibitor is acting. It is thus difficult to predict, at present, the overall clinical effect of a microglia depleting therapy in AD.

Although several anti CSF1R drugs have been developed and tested in different diseases, JNJ-40346527 is the only one that has entered clinical development in AD. In preclinical studies, in the prion mice, the drug showed brain permeability and inhibited proliferation of microglia (Mancuso et al., 2019). Although no published data are available in AD mouse models, in the P301S tauopathy mouse model, edicotinib significantly inhibited microglial proliferation, tau phosphorylation, neurodegeneration, and normalized the gene expression profile of microglia (Mancuso et al., 2019). A phase 1b trial was initiated in people with mild cognitive impairment, with a CDR global score of 0.5 and slight impairment in delay or free recall (NCT04121208). The study focuses on changes in CSF-1R signaling and microglia status in 54 participants randomized 2:1 to receive 300 mg JNJ-40346527 twice daily or placebo. Following 2 weeks of treatment, depending on the results, the trial is planned to continue into a second randomized phase, at a dose to be determined. Primary outcome is change in the concentration of CSF-1R ligands in cerebrospinal fluid. Secondary measures include unspecified biomarkers in CSF and plasma, CSF levels of microglia-derived extracellular vesicles and cells, CSF and plasma JNJ-40346527 levels, and safety assessments. The trial was due to end in 2021. No update on trial results is available on the clinicalstudies.gov site. CSF1R inhibitors have already been studied in several human diseases and JNJ-40346527 proved ineffective in Phase 2 trials for rheumatoid arthritis (Genovese et al., 2015), Crohn’s disease (Provention Bio press release, 2019), and Hodgkin’s lymphoma (von Tresckow et al., 2015). The availability of a large number of exposure data to CSF1R inhibitors allows a characterisation of the safety profile of this class of drugs, which appears not negligible. In particular, the CSF1R inhibitor Pexidartinib approved by the FDA for tenosynovial giant cell tumour (FDA pexidartinib), was rejected by the EMA due to questionable efficacy and observed hepatotoxicity (EPAR Turalio).

#### 5.5.4 Sargramostim

Sargramostim is a recombinant human GM-CSF, approved by FDA to accelerate bone marrow recovery in diverse settings of bone marrow insufficiency. GM-CSF was shown to activate microglia, reduce amyloid pathology by more than 50%, and reverse the cognitive impairment of transgenic AD mice (Boyd et al., 2010; Kiyota et al., 2018). Treatment with sargramostim was associated with improved cognition in cancer patients undergoing hematopoietic stem cell transplantation (Jim et al., 2012). In a placebo-controlled, randomized, double-blind Phase II clinical trial run by the University of Colorado in mild-to-moderate AD participants, subcutaneous injection of sargramostim (5 days/week for 3 weeks) was associated with reduced plasma levels of total tau (24%) and UCHL1 (42%), a biomarker of neuronal neurodegeneration, and improved cognition based on MMSE scores. At end of study treatment (EOT), the mean MMSE total score change in the sargramostim group was 1.45 units higher relative to baseline (*p* = 0.0074). The difference in mean change from baseline in MMSE total scores between the sargramostim and placebo groups was 1.80 (*p* = 0.0370) at EOT and 1.75 (*p* = 0.0272) at the first follow-up visit, 45 days after EOT, but disappeared by 90 days. The ADAS-Cog13 did not differ at end of treatment but was worse in the treated group at day 45 (Potter et al., 2021). The same authors are currently running a second phase II trial (NCT04902703) in 42 patients with mild to moderate Alzheimer, confirmed by CSF amyloid pathology, and a MoCa score of 10–20 inclusive, who will be treated with a 6-month course of the same dose of sargramostim given 5 days a week. The MoCa scale, differently from the MMSE, tests also for executive function (Dautzenberg et al., 2020), however, its use as a diagnostic instrument appears debatable with a study showing a positive predictive value for diagnosis of mild Alzheimer of only 31% (Dautzenberg et al., 2020). The primary endpoint of the study is safety, while clinical changes in terms of MMSE scoring are included as secondary endpoints.

Given the heterogenicity of microglia population and the present incomplete knowledge of the factors that drive the shifting among the different microglia phenotypes, it is difficult to select the most adequate patient population in which to test the two groups of drugs acting through CSF receptors: recombinant GM-CSF and CSF1R inhibitors. Early in AD course, activated microglia cluster around amyloid plaques, restricting their spreading to neurites. In this stage, GM-CSF may act to increase the microglia population with a protective phenotype, whereas CSF1R inhibitors may reduce the number of pro-inflammatory microglia in favour of the homeostatic phenotype. In later AD stages characterised by the spreading of NFT and neuroinflammation, CSF1R inhibitors could prevalently act by decreasing the number of microglia with a pro-inflammatory phenotype, whereas GM-CSF might exert favourable effects by their immunomodulatory action as suggested by sargramostim-induced increase in the levels of both inflammatory (IL-6 and TNF-α) as well as ant-inflammatory (Il-10) cytokines, observed in patients with mild to moderate AD (Potter et al., 2021).

#### 5.5.5 Daratumumab

Daratumumab is a human monoclonal antibody that targets CD38, approved by FDA and EMA for the treatment of multiple myeloma. CD38 is a NAD glycohydrolase expressed by neurons, astrocytes, microglial cells and CD8^+^ T cells. It regulates inflammation by degrading NAD, and by regulating calcium signaling and migration of inflammatory cells through the production of NAD-derived metabolites. CD38 expression increased after neuroinflammatory insults and CD38 siRNA knockdown reduced astrocyte pro-inflammatory cytokines and chemokines production (Kou et al., 2009). CD38 is also involved in astrocyte-induced neuroprotection as it participated to the transfer of mitochondria from astrocytes to neurons after stroke (Hayakawa et al., 2016).

In microglia CD38 plays a more complex, double-edged role. In LPS-stimulated microglia, CD38 knockdown reduced the release of inflammatory cytokines and favoured microglia survival, whereas its switch off in normal microglia resulted in increased apoptosis (Wang Y. M. et al, 2017). In AD, CD38 immunoreactivity was observed in NFTs (Otsuka et al., 1994). Indirect evidence of increased CD38 expression is provided by the observation of decreased levels of NAD (Sonntag et al., 2017), as well as by a decline in CD38 expression-inhibiting miRNAs in the CSF of AD patients compared to age-matched controls (Denk et al., 2015; Guerreiro et al., 2020).

Contrasting results were obtained by CD38 deletion in normal mice and in a mouse model of AD. While in normal mice CD38 KO resulted in deficits in various learning and memory tasks (Kim et al., 2016), in APPswePS1ΔE9 mice crossed with CD38 KO mice the Aβ burden was reduced, and spatial learning was improved (Blacher et al., 2015). Whether this apparent inconsistency may be linked to the double-edged role of CD38 in microglia is at present not known.

A recent paper by Gate et al. (2020), reported that the expression of CD38 is significantly increased on CD8^+^ T cells in the blood and the cerebrospinal fluid of early AD patients as compared with age-matched controls. In their rational for studying daratumumab in AD, Janssen do not include a direct effect of daratumumab on CNS resident cells, but highlight hypothesises that daratumumab potential effect in AD may be mediated by the inhibition of tissue invasion of blood cell-derived lymphocytes (NCT04070378). The Janssen’s proposed rational casts doubts on the extent of BBB permeability of daratumumab. This may be an issue potentially impacting on drug efficacy in AD, given that the relevance of the contribution of the adaptive immune system to AD is at present not known and could be far less important than the action of the innate immune cells (for a review see Heppner et al., 2015). Indeed, although in the literature the drug is always referred to as brain-permeable, measurements of daratumumab in CSF are scanty, and only two papers addressing this issue are retrievable online, one of which reports, in the CSF of a patient with Leptomeningeal Multiple Myeloma, a concentration of daratumumab 71 times lower compared to serum levels (Zajec et al., 2020). A phase II open-label pilot study is currently testing the potential clinical effect of daratumumab in AD. Fifteen patients with mild to moderate AD were selected based on MMSE score of 15–26 inclusive, and positive MRI and amyloid PET scans. Recruited subjects received daratumumab given s.c. at the dose of 1800 mg once weekly for 8 weeks followed by daratumumab s.c. 1800 mg every 2 weeks for 16 weeks. The primary endpoint is ADAS-cog/11. Patients with improvement of ≥4 points, 1 week after completing 24 weeks of treatment, will be considered responders. The choice of ADAS-cog/11 as primary outcome does not include executive function testing, which instead is important in mild to moderate AD patients. The ability of the primary measure to adequately reflect clinically relevant effects is thus limited, however a battery of scales measuring cognition as well functioning is included as secondary endpoint (ADAS-cog/12, MMSE, CDR-SB, ADCOMS). The definition of responders as achieving at least a 4-points improvement appears consistent with a progression rate of 5.5 11 points per year in ADAS-cog_11_ scale, in the mild to moderate AD historical population, which is generally well accepted in the published AD literature (Ito et al., 2010; Samtani et al., 2015). The safety profile of daratumumab as i.v. treatment in multiple myeloma is already fully characterised, and includes the possibility to trigger antibody-dependent cell-mediated cytotoxicity, and among adverse events listed as very common: respiratory tract infections, neutropenia and thrombocytopenia, peripheral sensory neuropathy and infusion reactions (see EPAR darzalex and FDA darzalex) However, in the ongoing phase II trial in AD (NCT04070378), daratumumab is administered s.c. and at a lower dose, which may ameliorate the safety profile. The study is estimated to be completed within June 2024.

#### 5.5.6 Pepinemab

Pepinemab (VX15/2503) is a monoclonal antibody that directly targets Semaphorin 4D/CD100, a glycoprotein that in the nervous system is expressed by both neuronal and glial cells (Alto and Terman, 2017; Lee et al., 2019). Semaphorins affect learning and memory by modulation of synaptic transmission and plasticity in the hippocampus (Lee et al., 2019; Zhang L. et al., 2021), are involved in tissue repair, and induce glial and endothelial cells’ activation, survival and migration, as well as immune cell regulation (Kumanogoh and Kikutani, 2004; Smith et al., 2015). In particular, SEMA4D was selected as a potential therapeutic target in Huntington disease (HD), multiple sclerosis (MS) and lastly AD. SEMA4D drove neuroinflammation by downstream activation of Rho GTPases, phospho-AKT and NF-kB signaling (Alto and Terman, 2017; Lee et al., 2019; Evans et al., 2022). Preclinical studies aimed at deciphering the exact role of SEMA4D in neuroinflammation in AD currently point to a main role for astroglial expression/responsiveness. SEMA4D knockout in astrocytes inhibited their ability to proliferate and become activated *in vitro* (Ben-Gigi et al., 2015) and reduced neuronal death from cortical injury in mice (Sweetat et al., 2022). In another study analysing brains from both post-mortem AD patients and AD murine models, SEMA4D was overexpressed in neuronal cells and activated astrocytes, expressing cognate receptor, were detected in their close proximity. These astrocytes displayed a disrupted homeostasis, including impaired glucose uptake and neurotransmitter recycling (Evans et al., 2022). A SEMA4D blocking antibody was reported to reduced astrogliosis, BBB impairment and pathology in rat models of experimental autoimmune encephalomyelitis (EAE) and produce beneficial effects on cognitive performances in the CVN mice, a disease model that reproduces many features of AD-like pathology, directinteraction with Plexin including neuroinflammation (Smith et al., 2015; Evans et al., 2022). More controversial is the interpretation of SEMA4D effects on microglial activation. In fact, the protein was shown to activate microglia and compromise the stability of the BBB in *in vivo* models of MS and hypoxia (Smith et al., 2015; Kuklina, 2019) whereas in other studies, SEMA4D was shown to mitigate LPS-induced microglial activation *in vitro* (Toguchi et al., 2009) and its silencing compromised neuronal recovery from spinal cord injury in the zebrafish model (Peng et al., 2017). According to recent findings from an EAE model, microglial SEMA4D mediated direct interaction with PlexinB receptors expressed by astrocytes, confirming converging astro-microglial signaling during neuroinflammation (Clark et al., 2021). Altogether, these aspects surely deserve a deeper investigation and could be relevant to better pinpoint SEMA4D-blockade-based therapeutic strategies. The brain permeable antibody Pepinemab (Evans et al., 2020; Feigin et al., 2022; Fisher et al., 2022) was first developed in 2016 as a SEMA4D-directed antibody hindering its binding to cognate PlexinB1/B2 receptor (Fisher et al., 2016). The “SIGNAL-AD” clinical trial, designed to test Pepinemab for therapy in early stages of AD, was started in mid-2021, after the publication of results from the phase 1/2 trial testing Pepinemab in HD (SIGNAL-HD/NCT04381468; Feigin et al., 2022; Zauderer and Evans, 2023). Despite the unmet primary endpoints, the SIGNAL-HD study suggested a positive effect of the drug on cognition and brain metabolic activity, based on which it is currently programmed to continue to phase 3. Since the inflammation-induced decline in glucose metabolism, associated with cognitive deficits, is a shared feature between HD and AD, results from the HD study provided a solid rationale for testing Pepinemab also in AD (Zauderer and Evans, 2023). It is worth noting that the beneficial cognitive effects of Pepinemab in HD were more pronounced in early manifest patients than prodromal patients (Feigin et al., 2022). This may seem in contrast with the idea that the earliest possible intervention is required to prevent/delay glial activation and neuroinflammation damage. In this case, a possible explanation is that cognitive amelioration over 18 months in prodromal patients was partly masked by the slow progression of clinical symptoms during earlier stages of HD (Feigin et al., 2022). The SIGNAL-AD randomized, double blind phase 1a/2b study is currently enrolling AD patients with mild dementia, positive for amyloid biomarkers (PET scan or CSF levels). In terms of cognitive impairment, the criteria for inclusion indicate probable AD with Global CDR of 0.5 or 1.0 and MMSE) score of 17–26. A total of 40 patients will be subjected to monthly IV infusions over 44 weeks of Pepinemab or placebo. The endpoints of the study will be the safety and tolerability of Pepinemab and the effects on cognition and brain metabolism, with an estimated primary completion date by the end of 2023. Multiple tests will be run for cognitive evaluation, including the ADAS-cog13, CDR and MMSE. Changes in brain metabolism and brain volume will also be determined. As for the safety data, both the HD and the MS clinical trials have so far shown a good safety profile and tolerance vs. placebo, although at lower doses of Pepinemab (LaGanke et al., 2017; Feigin et al., 2022). It is finally important to point out that preclinical data from a rat host resistance model excluded immunosuppressive effects by SEMA4D blocking antibody (Leonard et al., 2015).

### 5.6 Drug described in patents

The search in WIPO- IP Portal retrieved thirteen compounds that were patented based on their potential anti-inflammatory action in AD. Some of them fall within the category of natural products or their chemical derivatives, others are drugs already in use in the clinics with indications other than AD. Development of derivatives was always aimed at improving efficacy, target selectivity or CNS permeability. Preclinical research on these compounds was carried out *in vitro* and/or *in vivo* to assess the mechanisms of action and efficacy, although data appear sound for some compounds and still scarce for others. Based on their pharmacological action and exclusively taking into account the anti-inflammatory effects, the drugs can be grouped as follows: i) direct inhibitors of canonical pro-inflammatory pathways (Baohuoside I a.k.a icariside II; artemisinin B, genistein derivative DL0140-3; Achillea fragrantissima derivatives; butylphthalide-telmisartan heterocomplex; dapansutrile; CAP37/cathepsin G/neutrophil elastase peptides; acyclovir/dexamethasone); ii) agents targeting key enzymes/receptors involved in potentially detrimental biochemical pathways when deregulated (FLAP inhibitors; acid sphingomyelinase inhibitors; isoflavone compound J37941; α7 nicotinic receptor binding agents) and iii) compounds still in need of clarification (furanone derivatives). A description is provided in Supplememtary Table S2 in Data Sheet 1.

## 6 Conclusion

Accumulating new data show that activation of the immune system, and in particular innate immunity, plays a relevant role in the AD pathology. Targeting neuroinflammation may thus be a therapeutic strategy that can complement the recent approved immunotherapy with anti-Aβ antibodies, providing a multiple-front attack against the disease. In agreement, not only a significant amount of drugs targeting inflammation are at present in clinical trials, but also several drugs aimed at reducing inflammation have been recently patented for their potential use in AD, including natural compounds, synthetic derivatives, and repurposed drugs. Given the limited efficacy in slowing the progression of the disease observed with lecanemab, an average difference of 0.45 points on the primary endpoint CDR-SB, when the literature debate suggests a 1-point change minimum to be clinically relevant (Lansdall et al., 2023), it is reasonable to think that a combination therapy, targeting different actors involved in the pathology, could result in larger treatment benefit. In addition, combination therapy might allow the use of lower doses of each component, resulting in better treatment safety. A valuable result in view of the risk of hemorrhages observed with anti-Aβ antibodies (Honig et al., 2023).

Regulators (FDA and EMA) have endorsed the concept of combination therapies and have issued guidance for co-development of two or more new investigation drugs for use in combination (EMA guidelines Alzheimer disease; FDA co-development of combination therapies). However, combination therapy may be challenging for several reasons, including the complexity of clinical trial design, and need for cooperation between pharmaceutical industries. Regulators and payers are interested in having both the demonstration of additive or synergistic effects as well as the contribution of each clinical candidate in the combination to the overall effect. This requires much larger sample sizes than currently used in phase 2 and phase 3 studies. Moreover, cognitive measures lack the sensitivity to detect subtle changes quickly, especially in early stages of AD. Downstream functional markers are needed to help the evaluation of combination therapies. In case two companies are involved in the development of combination treatment, business-related issues such as intellectual property and data sharing add complexities to the picture. Consortia between industries and academia, and the use of existing adaptive platforms that enable parallel assessment of multiple drugs and treatment regimens, use of uniform protocols and outcome measures, and allow treatment arms to be added or dropped based on interim analyses of outcomes, could help to solve some issues (Aisen et al., 2021).

The optimization of such combined therapy is, in any case, complex, because the disease develops through a continuum of states that are at present only partially characterized and are endowed with significant variability in different patients. Thus, treatment probably needs to be fine-tuned. Several studies with drugs targeting neuroinflammation have revealed not only that the efficacy of anti-inflammatory therapies could be restricted to a certain time window in the disease course, which may be not easy to intercept in each patient, but also that some therapies, if given outside of their optimal timeframe, could be even detrimental. This may be true particularly for those treatments targeting CNS-resident microglia that transit through different phenotypes, protective or pro-inflammatory, overlapping each other during the course of the disease, but also for agents modulating kinase signaling that may have context-dependent effects due to the broad involvement of the targeted enzymes in brain physiology. Further complexity is added by the need to optimize the pharmacokinetics of the potential drug treatments. If the low level of peripheral inflammation that is observed in AD patients is ultimately recognized as only being an extra in the pathology, and the same is for recruitment of cells of adaptive immunity to the AD brain, new agents with high brain permeability need to be developed. Further insights in the role of the immune system along the course of AD, and the validation of biomarkers for a more effective stratification of patients are thus needed to design efficacious therapeutic combination strategies in AD.

## Footnotes

1 U.S. National Library of Medicine. ClinicalTrials.gov website [Internet]. Accessed 24 April 2023. Available from: https://clinicaltrials.gov.

2 World Intellectual Property Organization. WIPO IP Portal [Internet]. Geneva (CH); accessed 24 April 2023. Available from: https://patentscope.wipo.int/search/en/search.jsf.
