# Supplementary material for: Alzheimer’s disease and neuroinflammation: will new drugs in clinical trials pave the way to a multi-target therapy?
Source: Front Pharmacol. 2023 Jun 2;14:1196413. doi: 10.3389/fphar.2023.1196413 (PMC10272781; doi:10.3389/fphar.2023.1196413)
Supplement: Supplementary file 1 [file DataSheet1.pdf]

Supplementary Material

Alzheimer's disease and neuroinflammation: will new drugs in clinical trials pave the way to a multi-target therapy?

Daniela Melchiorri[1], Sara Merlo[2†], Benjamin Micallef[3†], John-Joseph Borg[3,4], František Dráfi[5,6]

†These authors contributed equally to this work and share second authorship

[1] Department of Physiology and Pharmacology, Sapienza University, Rome, Italy

[2] Department of Biomedical and Biotechnological Sciences, Section of Pharmacology, University of Catania, Italy

[3] Malta Medicines Authority, Sir Temi Żammit Buildings, Malta Life Sciences Park, San Ġwann SĠN 3000

[4] School of Pharmacy, Department of Biology, University of Tor Vergata, Rome, Italy

[5] Institute of Experimental Pharmacology and Toxicology, Centre of Experimental Medicine SAS Bratislava, Slovakia

[6] State Institute for Drug Control, Bratislava, Slovakia

*** Correspondence:** Daniela Melchiorri: daniela.melchiorri@uniroma1.it

1 Supplementary Tables

Table 1: Investigational Medicinal Products in development for neuroinflammation, mechanism, target population and endpoints.

Agents modulating microglia and astrocyte activation (N=6)

Active Substance<br>(Category)	Mechanism of Action	NCT number (Status) – Phase	Population Targeted Enrolment	Sponsor (Industry / Academic)	Design<br>Outcome measures	Trial Start / End
AL002<br>(Biological)	Monoclonal antibody targeting TREM2 receptors to promote microglial clearance of Aβ	NCT04592874 (Recruiting) - Phase II	MCI and mild AD<br>Brain amyloidosis by CSF or PET;<br>MMSE score ≥ 22 points, CDR Global Score of 0.5 - 1.0;<br>RBANS score on the DMI ≤85. (Enrolment 265 adults, older adults)	Alector Inc. & AbbVie (Industry)	Randomized, parallel assignment, quadruple blinded, placebo-controlled study to evaluate the efficacy and safety of AL002 in participants with early Alzheimer's disease.<br>Primary Endpoints: Disease progression as measured by the CDR-SB.<br>Secondary Endpoints: Change in MMSE score, Change in RBANS score, Change in ADAS-Cog13 score, Change in ADCS-ADL-MCI score, Change in ADCOMS score, Evaluation of safety and tolerability of AL002, Incidence of adverse events.<br>Other Endpoints: Change in brain volume measure by MRI, blood-based biomarker, CSF biomarkers, amyloid burden & tau tangle assessed by PET, Winterlight Language Speech Assessment score.	22 January, 2021 / Jan-24
		NCT05744401 (Recruiting) – Phase II	Same as NCT04592874 (parent study)<br>(Enrolment 190 adults, older adults)	Alector Inc. & AbbVie (Industry)	Randomized, parallel-group, long-term extension, dose-blind, multicentre study enrolling participants who completed NCT04592874 (parent study).<br>Primary Endpoints: Safety and tolerability (AEs, MRI abnormalities), Immunogenicity as measured by ADAs titters.	4 January, 2023 / Dec-2025

Active Substance<br>(Category)	Mechanism of Action	NCT number (Status) – Phase	Population Targeted Enrolment	Sponsor (Industry / Academic)	Design<br>Outcome measures	Trial Start / End
AL002<br>(Biological)		NCT03635047 (Completed – no results posted) – Phase I	Healthy adults<br>Mild to moderate AD based on NIA-AA criteria (Enrolment 69 adults, older adults)	Alector Inc. (Industry)	Randomized, parallel assignment, triple blind, placebo-controlled study evaluating the safety, tolerability, pharmacokinetics, pharmacodynamics, and immunogenicity of single and multiple doses of AL002 in Healthy Participants and in Participants with Mild to Moderate Alzheimer's Disease.<br>Primary Endpoints: Evaluation of safety and tolerability measured by number of subjects with AEs and Dose Limiting Adverse Event.<br>Secondary Endpoints: PK, C_{max}, AUC.	12 November 2018 / 25 November 2020
TB006<br>(Biological)	Monoclonal antibody targeting Galactin 3	NCT05074498 (completed) - Phase Ib/II	Mild to severe AD with MMSE score of 24 or less<br>(Enrolment 140 Adults, Older Adults)	TrueBinding Inc. (Industry)	Randomized, sequential multiple Dose-escalation assignment, triple blinded, Phase 1b/2a study evaluating Safety, Tolerability, Pharmacokinetics, Pharmacodynamics and Efficacy of TB006 in Patients with Mild to Severe Alzheimer's Disease.<br>Part 1 Primary Endpoints: Number of Participants with TEAE, Number of Participants with Clinically Significant Clinical Laboratory Parameter Values, Number of Participants with Clinically Significant Vital Sign Values, Number of Participants with Clinically Significant 12-Lead Electrocardiogram Findings, Change from Baseline in C-SSRS Scores, Number of Participants with Clinically Significant Physical Examination Findings, Number of Participants with Clinically Significant Neurological Examination Findings, Area under the Concentration Time Curve over a Dosing Interval (AUC_{tau}) of TB006, C_{max} of TB006, T_{max} of TB006, C_{trough} of TB006, t1/2 of TB006, CL of TB006, V_d of TB006, Concentrations of TB006 in CSF as a Measure of the Extent of CSF Distribution, Number of Participants with Anti-TB006 Antibodies.	8 October, 2021 / 15 October, 2022

**Active Substance (Category)**	**Mechanism of Action**	**NCT number (Status) – Phase**	**Population Targeted Enrolment**	**Sponsor (Industry / Academic)**	**Design Outcome measures**	**Trial Start / End**
TB006 (Biological)					Part 2 Primary endpoint: Change from Baseline through Day 104 on the CDR-SB Total Score<br><br>Secondary Endpoints: Change from Baseline through Day 36 on the CDR-SB Total Score, Number of Responders on the CDR-SB at Days 36 and 104, Change from Baseline to Days 36 and 104 on the Cognitive Drug Research System Battery, Composite Scores and Individual Task Measures, Change from Baseline to Days 36 and 104 on the MMSE Score, Change from Baseline to Days 36 and 104 on the NPI Score, Number of Participants with TEAE, Number of participants with Clinically Significant Clinical Laboratory Parameter Values, Number of Participants with Clinically Significant Vital Sign Values, Number of Participants with Clinically Significant 12-Lead Electrocardiogram Findings, Change from Baseline in C-SSRS Scores, Number of Participants with Clinically Significant Physical Examination Findings, Number of Participants with Clinically Significant Neurological Examination Findings, C_{trough} of TB006, C_{max} of TB006, t1/2 of TB006.	
		NCT05476783<br><br>(Active, not recruiting) – Phase II	Long term extension study in carry-on patients from NCT05074498 and de novo AD patients with MMSE score of 24 or less<br><br>(Enrolment 180 adults, older adults)	TrueBinding Inc. (Industry)	Multi-center Open-label, single group assignment Long Term Extension Study to Assess the Safety of TB006 in Patients Who Have Completed Protocol TB006AD2102 and in De Novo Patients with Alzheimer's Disease<br><br>Primary Endpoints: AEs & SAEs, Clinically Significant Clinical Laboratory Parameter Values, Clinically Significant Vital Sign Values, Clinically Significant 12-Lead electrocardiogram Findings, C-SSRS, Clinically Significant Physical Examination Findings, Clinically Significant Neurological Examination Findings, MRI abnormalities, C_{max}, Anti-TB006 antibodies.	14 September, 2022 / October 2024

Active Substance (Category)	Mechanism of Action	NCT number (Status) – Phase	Population Targeted Enrolment	Sponsor (Industry / Academic)	Design Outcome measures	Trial Start / End
TB006 (Biological)					Secondary Endpoints: CDR SB score, cognitive functioning, MMSE score, NPI score, EQ 5D 5L QoL, brain atrophy measured by MRI, amyloid plaque using PET imaging, brain volume using MRI.	
		NCT04920786 (Recruiting) – Phase I	Healthy adult subjects (Enrolment 48 adults)	TrueBinding, Inc. (Industry)	Single dose, Randomized, Sequential Assignment dose-escalation study to assess the safety, tolerability, pharmacokinetics and pharmacodynamics of TB006 in Healthy Subjects. Primary Endpoints: AEs, single dose PK profile, MTD. Secondary Endpoints: $AUC_{D0\text{-}D29}$, $AUC_{D0\text{-}\infty}$, C_{max}, T_{max}, t(1/2), CL, V_d, CSF, safety and tolerability (AEs), anti-TB006 antibodies.	1 June, 2021 / 1 January, 2023
Edicotinib a.k.a JNJ-40346527 (Small molecule)	Colony-stimulating factor-1 receptor (CSF-1R) inhibitor; attenuates microglial proliferation and neurodegeneration	NCT04121208 (Unknown, last verified Oct 2020 as Not yet recruiting) – Phase I	MCI CDR Global Score = 0.5; Hopkin's verbal learning task-revised (HVLT-R) - delay recall and/or free recall > 1 SD (Enrolment 54 Adults)	University of Oxford (Academic)	Randomised, single-blind, placebo-controlled (P-C) parallel-group trial with JNJ-40346527 in adults with MCI. Primary Endpoints: P-C change from baseline in CSF protein marker concentration levels. Secondary Endpoints: P-C change from baseline in CSF and blood biomarker concentration levels, P-C change from baseline in amount of CSF extracellular vesicles and cell population, Measurement of plasma/CSF JNJ-40346527 levels, Measurement of CSF protein marker concentration levels following different JNJ-40346527 doses, Occurrence of AEs during the study.	November 2020 / December 2021
Pepinemab (Biological)	Monoclonal antibody directed at semaphorin 4D to reduce inflammation	NCT04381468 (Recruiting) - Phase I/II	MCI and mild AD Global CDR of 0.5 or 1.0;	Vaccinex Inc., (Industry)	Multicenter, Randomized, parallel assignment, Quadruple Blind, Placebo-Controlled Safety and Biomarker Study in Early-AD.	22 July, 2021 / 28 February, 2023

**Active Substance**<br>**(Category)**	**Mechanism of Action**	**NCT number (Status) – Phase**	**Population Targeted Enrolment**	**Sponsor (Industry / Academic)**	**Design**<br>**Outcome measures**	**Trial Start / End**
			MMSE score of 17-26 (inclusive);<br><br>positive amyloid CSF result;<br><br>positive amyloid PET scan<br><br>(Enrolment 40 adults, older adults).	Alzheimer's Drug Discovery Foundation, Alzheimer's Association (Academic)	Primary Endpoints: Number of subjects with TEAEs.<br><br>Secondary Endpoints: Effects on brain metabolism, ADAS-cog13, CDR, MMSE, ADCS-ADL, ADCS-CGIC, NPI, Immunogenicity of pepinemab in serum.<br><br>Other endpoints: PK parameter (C_{max}, AUC, T1/2), Serum and CSF levels of neuroinflammatory cytokines, T- and B-Cell Quantitation by Flow Cytometry, Plasma and CSF concentration of Nfl and Aβ1-42/Aβ1-40, CSF levels of pepinemab, CSF concentrations of tau, p-tau and YKL-40, Cellular SEMA4D levels, total soluble SEMA4D levels, Effects on brain volume (measured by MRI).	
Sargramostim (Biological)<br><br>***Authorised in AML and transplantation ***	Granulocyte macrophage colony-stimulating factor (GM-CSF)	NCT04902703 (Recruiting) – Phase II	Mild AD or moderate AD (NIA-AA criteria) MoCA score of 10-22 inclusive<br><br>positive biomarker for brain amyloid pathology as shown by CSF assay<br><br>(Enrolment 42 adults, older adults)	University of Colorado, Denver (Academic)	Randomized, placebo-controlled, parallel assignment, triple blind study of Safety and Efficacy.<br><br>Primary Endpoints: Safety as measured by number of AEs by body system.<br><br>Secondary Endpoints: Mini-Mental State Examination.<br><br>Other Endpoints: ADAS-Cog13, CDR-SB, Trail Making Test - Part A, ADCS-ADL, MoCA, NPI, Verbal Fluency, FDG PET assessment of brain metabolism.	1 June, 2022 / July 2024
Daratumumab<br><br>(Biological)<br><br>***Authorised for Multipole Myeloma***	Daratumumab is an IgG1κ human monoclonal antibody (mAb) that binds to the CD38 protein expressed on the surface of cells; regulates microglial activity	NCT04070378 (Recruiting) - Phase II	Mild to moderate Alzheimer Disease (NIA-AA) criteria)<br><br>MMSE score ≥ 15 and ≤ 26<br><br>MRI consistent with AD	Northwell Health,<br><br>Janssen (Industry)	Single Group Assignment, Open label, Pilot study.<br><br>Primary Endpoints: ADAS-cog/11.<br><br>Secondary Endpoints: ADAS-cog/12, MMSE, CDR-SB, ADCOMS, Treatment Emergent Adverse Effects, Treatment Emergent Serious Adverse Effects.	6 November, 2019 / 1 June, 2024

Active Substance<br><br>(Category)	Mechanism of Action	NCT number (Status) – Phase	Population Targeted Enrolment	Sponsor (Industry / Academic)	Design Outcome measures	Trial Start / End
			Amyloid positive PET scan (Enrolment 15 adults, older adults)			

Agents targeting intracellular inflammatory kinase signalling (N=4)

Active Substance<br><br>(Category)	Mechanism of Action	NCT number (Status) – Phase	Population Targeted Enrolment	Sponsor (Industry / Academic)	Design Outcome measures	Trial Start / End
NE3107<br><br>(Small molecule)	Synthetic derivative of androstenetriol acts as MAPK-1/3 inhibitor; reduces proinflammatory NF*κ*B activation	NCT04669028 (Recruiting) – Phase III	Mild to Moderate AD, (NIA-AA, 2011 criteria of all cause dementia and probable AD)<br><br>CDR Standard Global Score of 1 to 2 (inclusive) i.e mild to moderate;<br><br>MMSE score of ≥14 and ≤24 at both Screening and Baseline visits<br><br>(Enrolment 316 adults, older adults)	BioVie Inc. (Industry)	Quadruple Blind, Randomized, Placebo Controlled, Parallel Group, Multicentre study of NE3107 in Subjects Who Have Mild to Moderate Probable Alzheimer's Disease.<br><br>Primary Endpoints: Change in ADAS Cog12, Change in ADCS CGIC.<br><br>Secondary Endpoints: ADCS-ADL, MMSE, Neuropsychiatric Index 12, ADCOMS, Homeostatic assessment of insulin resistance 2, fasting blood glucose, postprandial glucose excursions<br><br>Other endpoints: Resource Utilization in Dementia, volumetric magnetic resonance imaging (vMRI), cortical metabolic rate using fluorodeoxyglucose positron emission.	5 August, 2021 / October 2023
NE3107<br><br>(Small molecule)		NCT05227820 (Completed – No results posted) – Phase II	Alzheimer Disease<br><br>CDR score of 0.5 to 1 suggesting mild cognitive impairment to mild dementia;	BioVie Inc. (Industry)	Single group assignment, open label study.<br><br>Primary Endpoints: fMRI MRS Change, fMRI DTI-NODDI Change, fMRI Arterial Spin Labelling Change, fMRI resting BOLD Seed Change, fMRI NVR Change.<br><br>Secondary Endpoints: Clinical Dementia Rating Change as calculated from the Quick Dementia Rating Scale Change, MoCA Change, ADAS-Cog Change, MMSE Change, Glucose Serology/Metabolic Level Change.	19 January, 2022 / 20

Active Substance<br><br>(Category)	Mechanism of Action	NCT number (Status) – Phase	Population Targeted Enrolment	Sponsor (Industry / Academic)	Design<br>Outcome measures	Trial Start / End
			one abnormal imaging biomarker (Enrolment 25 adults, older adults)			August, 2022
MW150<br><br>(Small molecule)	p38α MAPK Inhibitor, reduces inflammatory cytokines	NCT05194163 (Not yet recruiting)- Phase IIa	Mild to Moderate Alzheimer's Disease<br><br>MMSE score of 14 to 28;<br><br>CDR Global score of 0.5 to 2.0 inclusive<br><br>(Enrolment 24 adults, older adults)	Neurokine Therapeutics (Industry)<br><br>Columbia Univ.<br><br>National Institute on Aging (Academic)	Double-blind Randomized, Placebo Controlled, Parallel Group study.<br><br>Primary Endpoint: drug safety.<br><br>Secondary Endpoints: Cognitive change, MMSE, ADAS-cog, Executive, Language.<br><br>Functional performance- ADCS-ADL, CDR, NPI-Q, Pharmacodynamics – cytokines and neuronal biomarkers.	1 May, 2022 / 30 November, 2024
VX-745 a.k.a Neflamapimod<br><br>(Small molecule)	p38α MAPK Inhibitor, reduces inflammatory cytokines	NCT03402659 (Completed – Has results) – Phase II	MCI or Mild Alzheimer's Disease<br><br>CDR-Global Score of 0.5 or 1.0, with CDR memory sub score of at least 0.5;<br><br>MMSE score ranging from 20 to 28 (inclusive);<br><br>a CSF Aβ1-42R below the threshold and p-tau above the threshold;<br><br>CT or MRI findings compatible with AD (Enrolment 161 adults, older adults)	EIP Pharma Inc (Industry)	Double-blind, Randomized, Placebo Controlled, Parallel Group proof of concept study.<br><br>Primary Endpoints: Total and Delayed Recall on the HVLT-R.<br><br>Secondary Endpoints: Wechsler Memory Scale (WMS) Immediate and Delayed Recall, CDR-SB, MMSE, CSF total Tau, CSF p-tau, CSF AB1-40, CSF AB1-42, CSF Neurogranin, CSF Neurofilament Light Chain, P-tau/AB1-42 Ratio.	29 December, 2017 / 31 July, 2019

**Active Substance**<br><br>**(Category)**	**Mechanism of Action**	**NCT number (Status) – Phase**	**Population Targeted Enrolment**	**Sponsor (Industry / Academic)**	**Design Outcome measures**	**Trial Start / End**
VX-745 a.k.a Neflamapimod<br><br>(Small molecule)		NCT03435861 (Unknown – last verified in Feb 2021 as recruiting) – Phase II	MCI or Mild Alzheimer's Disease<br><br>MMSE > 20, Free and Cued Selective Reminding test < -1.5 DS<br><br>Documented cerebral amyloidopathy using CSF analysis or PET amyloid imaging<br><br>(Enrolment 40 Adults, Older Adults)	University Hospital, Toulouse (Academic)	Mono-centric, double-blinded, randomized, parallel assignment, placebo-controlled study assessing the effect of neflamapimod on brain inflammation in patients suffering of AD.<br><br>Primary Endpoints: brain inflammation assessed by [18F]-DPA714, standard uptake value in 1) Cortical region, 2) five lobes, 3) orbitofrontal, anterior cingulate, posterior cingulate and precuneus.<br><br>Secondary Endpoints: Neuropsychological assessment of memory, language and Attention and executive functions, Blood and CSF biomarkers of inflammation [ApoE phenotype, TSPO phenotype, TNFα, IL-1β, IFNγ, IL-12, IFNα/β, IL-10, IL-6, IL-8, MCP-1, IL-27, chemokines receptors, PD-1, p-tau, Aβ42, Aβ40, cells count, GM-CSF, PD-1, CD14/16].	8 October, 2018/ 30 June, 2021
Baricitinib<br><br>(Small Molecule)<br><br>***Authorised for RA***	Selective and reversible inhibitor of Janus kinase (JAK)1 and JAK2	NCT05189106 (Recruiting) – Phase I/II	Amyotrophic Lateral Sclerosis, Alzheimer Disease, Mild Cognitive Impairment<br><br>CSF level of CCL2 level ≥ 250 pg/mL<br><br>For AD: MoCA score ≥ 8<br><br>(Enrolment 20 Adults, Older Adults)	Massachusetts General Hospital (Academic)	Open label, Single group assignment, Proof of Concept basket Trial.<br><br>Primary Endpoints: CSF Concentration of baricitinib, CSF CCL2 Concentration.<br><br>Secondary Endpoints: CSF protein-kinase R (PKR), CSF phospho-PKR (pPKR), CSF pPKR/PKR ratio Concentration, CSF C-X-C motif chemokine ligand 10 (CXCL10) Concentration, CSF interferon gamma (IFNG) Concentration, CSF interleukin-6 (IL-6) Concentration, TAR DNA-binding protein 43 (TDP-43) Plasma Levels, CSF neurofilament light chain Concentration, CSF tau Concentration, CSF phospho-tau (pTau), Incidence of AEs.	5 December, 2022 / 1 October, 2024

Agents inhibiting the action or the production of pro-inflammatory cytokines or eicosanoids (N = 6)

Active Substance (Category)	Mechanism of Action	NCT number (Status) – Phase	Population Targeted Enrolment	Sponsor (Industry / Academic)	Design Outcome measures	Trial Start / End
XPro1595 a.k.a Pegipanermin<br><br>(Biological)	TNF inhibitor; reduces neuroinflammation mediated by TNF receptor type 1	NCT03943264 (Completed – No results posted) - Phase I	Alzheimer Disease<br><br>Diagnosed with probable AD defined by the NINCDS-ADRDA criteria<br><br>hsCRP levels ≥1.5mg/L, OR HbA1c ≥ 6DCCT %, OR ESR ≥10 mm/h, OR APOE4 positive (at least one APOE4 allele)<br><br>(Enrolment 20 adults, older adults)	Inmune Bio, Inc. (Industry)	Non-Randomized, Parallel Assignment, Open Label study.<br><br>Primary Endpoints: The number / percentage of patients with a TEAE throughout 12 weeks of treatment with XPro1595.<br><br>Secondary Endpoints: Changes from baseline in high sensitivity C-reactive protein in the blood and CSF, Changes from baseline in inflammatory cytokines in the blood and CSF, Changes from baseline in blood and CSF levels of amyloid beta, Changes from baseline in CSF levels of tau, Change from baseline in FreeWater content (oedema) using MRI, Change from baseline in the MMSE, Change from baseline in the DSST, Change from baseline in the Verbal Fluency Test, Change from baseline in the NPI, Change from baseline in the Bristol ADL Scale, Change from baseline in the MERET OBSRO-C, Evaluate changes in the MERET PGI-C [all secondary endpoints measured at 12 weeks].<br><br>Other Endpoints: Change from baseline in Breath volatile organic compounds.	20 November, 2019 / 1 September, 2021
XPro1595 a.k.a Pegipanermin<br><br>(Biological)		NCT05318976 (Recruiting) - Phase II	Mild Alzheimer's Disease with inflammation<br><br>mild dementia as clinically described in McKhann, (2011) and corresponding to stage 4 of the revised AD	Inmune Bio, Inc. (Industry)	Randomized, Placebo-Controlled, Double-Blind Study of XPro1595 in Patients with Mild Alzheimer's Disease with Biomarkers of Inflammation.<br><br>Primary Endpoints: Change in EMACC.<br><br>Secondary Endpoints: Change in CDR, Change in apparent fiber density, Change in E-Cog, Change in ADCS-ADL, Change in myelin content, Change in non-cognitive behavioural symptoms, Change in gray matter integrity, Change in blood inflammatory and	28 February, 2022 / 19 June, 2023

Active Substance (Category)	Mechanism of Action	NCT number (Status) – Phase	Population Targeted Enrolment	Sponsor (Industry / Academic)	Design Outcome measures	Trial Start / End
			staging system (Jack, 2018)<br><br>Amyloid positive<br><br>(Enrolment 201 adults, older adults)		neurodegeneration biomarkers (on blood inflammatory and neurodegeneration biomarker amyloid), Change in blood inflammatory and neurodegeneration biomarkers (on blood inflammatory and neurodegeneration biomarker pTau), Change in brain structure neurodegeneration, Number of participants who experience AEs and SAEs.	
XPro1595 a.k.a Pegipanermin<br><br>(Biological)		NCT05321498 (Not yet recruiting) –<br><br>Phase II	MCI<br><br>Diagnosed with MCI of probable Alzheimer's disease (Albert 2011; NIA-AA]<br><br>Amyloid positive<br><br>(Enrolment 60 adults, older adults)	Inmune Bio, Inc. (Industry)	Randomized, Parallel assignment, Quadruple blinded, Placebo-Controlled, Study of XPro1595 in Patients with MCI With Biomarkers of Inflammation.<br><br>Primary Endpoints: Change in EMACC.<br><br>Secondary Endpoints: Change in Mean Computer-based Cognitive Assessment (Cogstate) Composite score from Screening to Week 12, Change in myelin content, Change in blood inflammatory and neurodegeneration biomarkers (amyloid & pTau), Change in Imaging (MRI) Neuroinflammation, Change in imaging markers of brain quality, Change in apparent fiber density, Change in non-cognitive behavioural symptoms, Change in brain activity, Change in speech and language, Change ADCS-ADL-MCI, Change in CDR-SB, Number of participants who experience AEs & SAEs.	September 2022 / 26 January, 2023

**Active Substance (Category)**	**Mechanism of Action**	**NCT number (Status) – Phase**	**Population Targeted Enrolment**	**Sponsor (Industry / Academic)**	**Design Outcome measures**	**Trial Start / End**
XPro1595 a.k.a Pegipanermin<br><br>(Biological)		NCT05522387 (Recruiting) – Phase II	Mild Alzheimer's Disease or MCI<br><br>who completed NCT05321498 or NCT05318976<br><br>(Enrolment 261 adults, older adults)	Inmune Bio, Inc. (Industry)	Single Group Assignment, Open-Label Extension of XPro1595 in Patients with AD or MCI to investigate safety, tolerability, and efficacy.<br><br>Primary Endpoints: AEs and SAEs.<br><br>Secondary Endpoints: Change in cognitive performance, change in cognition and global function, change in non-cognitive behavioural symptoms, change in ADL, change on blood inflammatory and neurodegeneration biomarkers [amyloid & pTau], change on imaging neuroinflammation [MRI White Matter Free Water], MRI Apparent Fiber Density, ECog.	November 2022 / 4 December, 2025
Canakinumab<br><br>(Biological)<br><br>***Authorised for various inflammatory diseases***	Canakinumab is a human monoclonal anti-human interleukin-1 beta (IL-1 beta) antibody of the IgG1/κ isotype. Canakinumab binds with high affinity specifically to human IL-1 beta and neutralises the biological activity of human IL-1 beta by blocking its interaction with IL-1 receptors, thereby preventing IL-1 beta-induced gene activation and the production of	NCT04795466 (Recruiting) - Phase II	MCI due to AD or mild AD (NIA-AA criteria)<br><br>CSF amyloid and tau positivity;<br><br>MMSE total score of 20 to 24 (inclusive) at screening; OR, MMSE total score of 25-30 (inclusive) plus a DSST score at least 0.5 SD below normative data at screening.<br><br>(Enrolment 90 adults, older adults)	Novartis (Industry)	Randomized, parallel assignment, quadruple blinded, exploratory platform study evaluating Efficacy, Safety, Tolerability and Pharmacokinetics.<br><br>Primary Endpoints: Change from baseline in cognition as measured by the Neuropsychological Test Battery (NTB) total score.<br><br>Secondary Endpoints: Number of participants who experience adverse events and serious adverse events, Change from baseline in microglia activation as measured by Positron-Emission Tomography-Translocator Protein 18kDa - microglia activation, change from baseline in neuropsychiatric symptoms as measured by the NPI total score, Change from baseline in function (ADL) as measured by the eCog total score, Change from baseline in memory as measured by the total Neuropsychological Test Battery memory composite score and change from baseline in executive function as measured by the total Neuropsychological Test Battery executive function composite score,	28 October, 2021 / 26 February, 2026

Active Substance (Category)	Mechanism of Action	NCT number (Status) – Phase	Population Targeted Enrolment	Sponsor (Industry / Academic)	Design Outcome measures	Trial Start / End
	inflammatory mediators.				Change from baseline in pharmacokinetic concentrations and immunogenetic anti-agent antibody levels in serum and/or plasma and/or CSF.	
Lenalidomide<br><br>(Small molecule)	Immunomodulatory agent that reduces inflammatory cytokines; modulates innate and adaptive immune responses	NCT04032626 (Recruiting) – Phase II	Mild Cognitive Impairment due to AD<br><br>Amnestic MCI (NIA-AA criteria) and with 1 of the following: MRI with hippocampal volume in the 5th percentile or lower for age, Amyloid PET positive at SUVr ≥ 1.05, CSF Tau profile with ATI lower than 1.0, FDG PET showing hypometabolism in the parietal temporal regions, or genetic confirmation of APOE4 (heterozygous or homozygous).<br><br>(Enrolment 30 adults, older adults)	St. Joseph's Hospital and Medical Center, Phoenix (Academic)	Monocentric, Randomized, Parallel Assignment, quadruple blind study evaluating Safety, Tolerability, and Efficacy.<br><br>Primary Endpoints: Change in cognition as assessed by the ADAS-Cog total score, change in cognition as assessed by the ADCS-ADL total score, Change in cognition as assessed by the CDR-SB total score, Change in cognition as assessed by the MMSE total score.<br><br>Secondary Endpoints: AEs % SAEs, Change in brain amyloid loads, change in neurodegeneration, Change in blood inflammatory markers.	22 July, 2020 / September 24

Active Substance (Category)	Mechanism of Action	NCT number (Status) – Phase	Population Targeted Enrolment	Sponsor (Industry / Academic)	Design Outcome measures	Trial Start / End
Emtricitabine<br><br>(Small molecule)<br><br>***Authorised for prevention and treatment of HIV***	Nucleoside reverse-transcriptase inhibitor – Decreases neuroinflammation	NCT04500847 (Recruiting) – Phase I	MCI and mild dementia due to AD (NIA-AA criteria)<br>MMSE 15-30 inclusive<br>CDR 0.5 - 2<br>CSF pTau/Aβ42 ratio of > 0.024<br>(Enrolment 35 adults, older adults)	Butler Hospital (Academic)	Randomized, Parallel Assignment, Quadruple blind, study.<br><br>Primary Endpoints: Number of participants with TEAEs in the treatment group will be compared to the placebo group.<br><br>Secondary Endpoints: Change from baseline in key inflammatory biomarkers; Tumor necrosis factor-alpha, Interleukin 1-beta, and Interferon-alpha, Change in MMSE Total Scores, Change from baseline in CDR, Change from baseline in ADAS-Cog -13, Change from baseline in ADCS-ADL, Change from baseline in Free and Cued Selective Reminding Test with delayed recall, Change from baseline in phosphorylated tau / amyloid beta 42 ratios.	17 December, 2021 / August 2023
Montelukast (small molecule)	Cysteinyl leukotriene type 1 (cysLT-1) receptor antagonist; effects on inflammatory processes, neuronal injury, blood-brain-barrier integrity, and A*β* protein accumulation	NCT03402503 (Recruiting) – Phase II	Mild to Moderate Alzheimer Disease<br><br>MMSE score of 14 - 22<br>CT or MRI indicating clinical phenotype of AD<br><br>(Enrolment 70 adults, older adults)	IntelGenx Corp. (Industry)	Multi-center, randomized, Parallel Assignment, triple blind, placebo-controlled study to Assess the Safety, Feasibility, Tolerability, and Efficacy.<br><br>Primary Endpoints: Global Neuropsychological test battery Composite.<br><br>Secondary Endpoints: MMSE, ADCS-CGIC, ADCS-ADL23, NPI, Sheehan Suicide Tracking Scale, Incontinency Frequency Rating, Incidence of TEAEs.	26 November, 2018 / 19 December, 2023
		NCT03991988 (Completed – No results posted) - Phase II	MCI and Early AD<br><br>For MCI group:<br><br>Abnormal memory function documented using the Logical	Emory University (Academic)	Randomized, Parallel Assignment, Double blind study.<br><br>Primary Endpoints: Number of participants with any gastrointestinal symptoms, Number of participants with reported anaphylaxis, Number of participants with	25 September, 2019 / 18 November, 2022

Active Substance (Category)	Mechanism of Action	NCT number (Status) – Phase	Population Targeted Enrolment	Sponsor (Industry / Academic)	Design Outcome measures	Trial Start / End
Montelukast (small molecule)			Memory subscale (Delayed Paragraph Recall, Paragraph A only) from the Wechsler Memory Scale-Revised, MoCA < 26; CDR scale /Memory box score=0.5; General functional performance sufficiently preserved (Functional Assessment Questionnaire ≤5).<br><br>For Early AD: Abnormal memory function documented using the Logical Memory subscale (Delayed Paragraph Recall, Paragraph A only) from the Wechsler Memory Scale-Revised; MoCA <26; CDR scale/Memory box score 1 or 2; Early AD dementia defined as Functional Assessment Staging Test of 4 or 5<br><br>(Enrolment 32 adults, older adults)		elevated liver enzymes, change in prothrombin time / international normalized ratio, Change in NPI-Q, Number of patients with seizures, Number of discontinuations from Montelukast.<br><br>Secondary Endpoints: Change in CSF amyloid, Change in CSF tau, Change in CDR, Change in NIH Toolbox Cognition battery.	

**Active Substance (Category)**	**Mechanism of Action**	**NCT number (Status) – Phase**	**Population Targeted Enrolment**	**Sponsor (Industry / Academic)**	**Design Outcome measures**	**Trial Start / End**
Salsalate<br><br>(Small molecule)	NSAID; anti-inflammatory and antirheumatic agent; inhibition of synthesis and release of prostaglandins	NCT03277573 (Unknown – last verified in March 021 as Active, not recruiting) – Phase Ib	Mild to Moderate Alzheimer Disease as per NIA-AA criteria<br><br>MRI at Screening is consistent with AD (≤ 4 microhaemorrhages, and no large strokes or severe white matter disease); MHIS at Screening is ≤ 4; MMSE at Screening is between 14 and 30 (inclusive)<br><br>(Enrolment 40 adults, older adults)	University of California, San Francisco (Academic)	Randomized, triple-Blind, parallel assignment, Placebo-Controlled Study evaluating Safety, Tolerability, Pharmacokinetics, Pharmacodynamics, and Preliminary Efficacy.<br><br>Primary Endpoints: Incidence of TEAE.<br><br>Secondary Endpoints: Changes in Pharmacokinetic properties of Salsalate in Plasma and CSF, Changes in Pharmacodynamic properties of Salsalate in CSF (CSF concentrations of total tau, phosphorylated tau, and neurofilament light chain), Change in brain volume on brain MRI, Change in structural and functional connectivity on brain MRI, Change in CSF Biomarkers of phosphorylated tau, Change in Cerebrospinal Fluid Biomarkers of NfL, Change in CSF Biomarkers of total tau, Change in Cerebrospinal Fluid Biomarkers of beta amyloid 1-42, Change in ADAS-Cog, Change in MMSE, Change in Alzheimer's disease Clinical Activities of Daily Living Scale, Change in CDR-SB.<br><br>Other Endpoints: Change in brain volume on brain MRI, Connectivity between brain regions measured using diffusion tensor MRI and resting state functional MRI, Cerebrospinal Fluid Biomarkers [p-tau, NfL, t-tau, Abeta 1-42], ADAS-cog, MMSE, ADCS-ADL, CDR-SB.	21 July, 2017 / 31 December, 2021

Active Substance (Category)	Mechanism of Action	NCT number (Status) – Phase	Population Targeted Enrolment	Sponsor (Industry / Academic)	Design Outcome measures	Trial Start / End
ALZT-OP1<br><br>(Small molecule)	Combination of anti-inflammatory drugs: Cromolyn (Cromone) and Ibuprofen (NSAID)	NCT02547818 (Completed – No results posted) – Phase III	Early AD<br><br>Logical Memory II subscale (Delayed Paragraph Recall) from the Wechsler Memory Scale<br><br>CDR (Global) = 0.5; Memory Box score must be at least 0.5<br><br>CSF Aβ-42 levels ≥ 180 pg/mL and ≤ 690 pg/mL; (Enrolment 620 adults, older adults)	AZTherapies, Inc. (Industry)	Randomized, Factorial Assignment, Quadruple masked, Safety and Efficacy study.<br><br>Primary Endpoints: CDR-SB.<br><br>Secondary Endpoints; Number of TEAE.	15 September, 2015/ 18 November, 2020
		NCT04570644 (Completed – No results posted) – Phase I/II	PK (Part A) n=24, both healthy volunteers and AD subjects<br><br>PD (Part B) n=32, AD subjects only mild to moderate Alzheimer's disease; Clinical Dementia Rating (Global) 0.5, Mini-mental state examination (MMSE) ≤ 22 (Enrolment 56 adults, older adults)	AZTherapies, Inc. (Industry)	Randomized, open-label, cross-over assignment, PK/PD and safety study.<br><br>Primary Endpoints: PK profile ($AUC_{0-\infty}$, AUC_{0-t}, AUC_{PLASMA}/AUC_{CSF}, CL/F C_{max}, t1/2, T_{max}, V_d/F.<br><br>Secondary Endpoints: Biomarker Beta Amyloid Sample Analysis plasma and CSF (Aβ-42, Aβ -40, Aβ-38), total Tau, NfL, Glial Fibrillary Acidic Protein, P-Tau, Interferon-γ (IFN-γ), TNF-α, TGF-β1, CD33, TREM2, Neurogranin.<br><br>Other Endpoints: TEAE.	28 August, 2020 / 18 January, 2021

Agent exerting a broad ranging immunomodulatory effect (N=1)

**Active Substance (Category)**	**Mechanism of Action**	**NCT number (Status) – Phase**	**Population Targeted Enrolment**	**Sponsor (Industry / Academic)**	**Design Outcome measures**	**Trial Start / End**
GB301 / VT301 (ATMP)	Autologous Regulatory T cells (‘Tregs’) control the inflammatory response of activated immune cells, reduce neuroinflammation, and thereby protect neurons.	NCT03865017 (Unknown – last verified in Feb 2019 as Not yet recruiting) - Phase I/II	Mild to moderate Alzheimer Disease (NIA-AA 2011 criteria)<br><br>MMSE ≥11<br><br>amyloid PET positivity<br><br>(Enrolment 20 Adults, Older Adults)	VTBIO Co. LTD (Industry)	Randomized, Parallel assignment, Quadruple blinded study single dose study.<br><br>Primary Endpoints: Number of subjects with Clinically Significant Abnormalities in 12-lead Electrocardiogram, Number of subjects with abnormal clinical chemistry parameters, Number of subjects with abnormal Hematology parameters, Number of subjects with abnormal Coagulation parameters, Number of subjects with abnormal Urinalysis parameters, Number of subjects with abnormal vital signs, Number of subjects with AEs, Number of subjects with abnormal physical examination, C-SSRS.<br><br>Secondary Endpoints: Change from Baseline in ADAS-Cog-13 Score, Change from baseline in MMSE score.	1 December, 2019 / 1 December, 2021
GB301 / VT301 (ATMP)		NCT05016427 (Recruiting) – Phase I	Mild to moderate Alzheimer Disease (NIA-AA criteria)<br><br>MMSE ≥ 10 points<br><br>CDR Global Score (CDR-GS) of 0.5 to 2.0 points<br><br>(Enrolment 12 Adults, Older Adults)	VTBIO Co. LTD (Industry)	Non-Randomized, Parallel Assignment, Open Label Dose-Escalating Study to Determine the Safety, and Tolerability.<br><br>Primary Endpoints: All AEs that occurred from the time of acquisition of consent of the subjects to the time of End of Study, Number of subjects with abnormal clinical Physical examination, Number of subjects With Clinically Significant Abnormalities in 12-lead Electrocardiogram, Number of subjects with abnormal clinical Laboratory Tests, Number of subjects with abnormal vital signs, Change from Screening "Questionnaire: C-SSRS" at 90 days.<br><br>Secondary Endpoints: Change from Baseline "Questionnaire ADAS-Cog-13", Change from Baseline	1 November, 2020 / 1 April, 2022

					"Questionnaire: ADCS-ADL", Change from Screening "Questionnaire: MMSE", Change from Screening "Questionnaire: CDR" [secondary endpoints at 90 days.].	

Table 2: Summary of drugs patented for Neuroinflammation in Alzheimer's disease

Direct inhibitors of canonical pro-inflammatory pathways (N=8)

Patent Details	Drug	Mechanism of Action (Neuroinflammation)/grounds for activity in neuroinflammation	Data from preclinical AD models	Comments/Perspectives
CN104173361: APPLICATION OF BAOHUOSIDE I IN PREPARATION OF MEDICINES FOR PREVENTING AND TREATING ALZHEIMER DISEASE<br><br>**Int.Class:** A61K 31/7048<br><br>**Appl.No:** 201410254624.6<br><br>**Applicant:** ZUNYI MEDICAL UNIVERSITY<br><br>**Inventor:** GONG QIHAI<br><br>Published on 03.12.2014	Baohuoside I (a.k.a Icariside II) (flavonoid) extracted from the traditional Chinese medicinal herb Epimedium brevicornum, endowed with anti-inflammatory, anti-oxidant, anti-cancer and cardioprotective properties (Xu et al., 2021a).<br><br>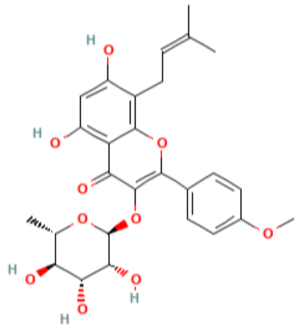<br>Pubchem	Icariside II was shown to possess phosphodiesterase 5 (PDE5) inhibiting activity (Li et al., 2022a). Notably, PDE5 inhibitors have been proposed as novel agents against neurodegeneration and have shown potential for rescue of memory disorders through activation of the NO/cGMP/PKG/CREB signalling pathway (Liu et al., 2019; Ribaudo et al., 2020; Zuccarello et al., 2020; Lee et al., 2022). A large amount of evidence from AD animal studies supported the efficacy of PDE5 inhibitors, as sildenafil and tadalafil, in reversing cognitive impairment and improving learning and memory, justifying the repurposing of this class of drugs in neurodegenerative diseases (Cuadrado-Tejedor et al., 2011; García-Barroso et al., 2013). However, in another recent study, the beneficial effects of PDE5 inhibitors in AD was not confirmed (Desai et al., 2022).	Icariside II was shown effective in vivo against cognitive deficits induced by Aβ25-35 injection and in vitro against Aβ-induced neurotoxicity. The mechanism involved activation of the BDNF/TrkB/CREB signalling cascade (Liu et al., 2018a). In another transgenic mouse model, icariside II proved effective in rescuing cognitive deficits both by affecting the amyloidogenic pathway and reducing inflammation via PPAR γ enhancement (Yan et al., 2017). In streptozotocin-induced mouse models of AD, icariside II prevented activation of NF-kB and release of inflammatory cytokines, providing cognitive protection (Yin et al., 2016). Direct anti-inflammatory effects of icariside II on both astrocytes and microglia were reported in LPS-induced inflammation models both in vitro and in vivo. The drug acted through inhibition of the TLR4/MyD88/NF-κB pathway and reduction of pro-inflammatory cytokines (Zhou et al., 2019; Zheng et al., 2020a). Recently, icariside II was also shown to rescue impaired	Icariside II represents a promising candidate drug for targeting inflammation in AD, given the evidence of a direct effect on glial cells and thanks to the potential cognitive benefits linked to its PDE5 inhibitor activity. A drawback to the potential use of icariside II as a therapeutic agent is its low oral bioavailability due to its poor aqueous solubility (Hou et al., 2016), which urges for the development of an improved formulation. Furthermore, given the broad biological actions resulting from PDE5 inhibition, potential side effects must be carefully taken into account.

Patent Details	Drug	Mechanism of Action (Neuroinflammation)/grounds for activity in neuroinflammation	Data from preclinical AD models	Comments/Perspectives
			neurogenesis and mitochondrial damage in AD mice, with a beneficial impact on cognition, through activation of the Wnt/GSK-3β pathway (Xiao et al., 2022).	
CN107802621: APPLICATION OF ARTEMISININ B TO RESISTANCE ON NEUROINFLAMMATION AND TREATMENT ON NEURODEGENERATIVE DISEASES<br><br>**Int.Class:** A61K 31/365<br><br>**Appl.No:** 201711272956.7<br><br>**Applicant:** INSTITUTE OF CHINESE MATERIA MEDICA, CHINA ACADEMY OF CHINESE MEDICAL SCIENCES<br><br>**Inventor:** ZHU XIAOXIN<br><br>Published on 16.03.2018	Artemisinin B is a sesquiterpene lactone extracted from *Artemisia annua L.*, well established for malaria therapy.<br><br>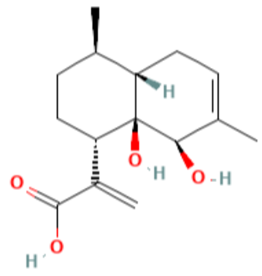<br><br>Pubchem	Artemisinin-type drugs have additionally been shown to be therapeutically effective in *in vivo* models of a wide variety of pathological conditions, all sharing a common neuroinflammatory component. These include rheumatic diseases (rheumatoid arthritis, osteoarthritis, lupus erythematosus, arthrosis, and gout), lung diseases (asthma, acute lung injury, and pulmonary fibrosis), skin diseases (dermatitis, rosacea, and psoriasis), neurological conditions (autoimmune encephalitis and myasthenia gravis), inflammatory bowel disease, and other inflammatory and autoimmune diseases (Efferth and Oesch., 2021; Arthur et al., 2022).	A number of *in vitro* and *in vivo* studies showed that artemisinin B was able to i) reduce the release of inflammatory cytokines, ii) inhibit NO, NF-κB, TLR4 and NLRP3-inflammasome signalling, iii) stimulate the ERK/CREB neuronal pathway, iv) reduce the deposition of Aβ and tau proteins and v) improve cognitive functions (Shi et al., 2013; Qiang et al., 2018; Zhao et al., 2020; Zhao et al., 2022b).<br><br>Very recent studies showed that artesunate, an artemisinin derivative, has similar anti-inflammatory effects on *in vitro* and *in vivo* AD models (Qin et al., 2022; Kuhse et al., 2023) and displayed an additional mechanism through increase of endothelial PICALM, a protein involved in Aβ clearance across the BBB and shown to be reduced in AD patients (Kisler et al., 2023).	Artemisin B appears promising as a modulating agent able to interfere with chronicization of inflammation, the most distinctive feature in AD progression, acting on multiple targets at once. However, the safety profile of this class of drugs may be an issue, particularly with long-term use needed for AD therapy. Artemisinin drugs are currently approved only for the acute treatment of malaria. Adverse events observed in this indication include very frequent gastrointestinal side effects, headache and dizziness, but also less frequent palpitation, QT prolongation, haemoglobinuria and acute renal failure.

Patent Details	Drug	Mechanism of Action (Neuroinflammation)/grounds for activity in neuroinflammation	Data from preclinical AD models	Comments/Perspectives
CN108236610: USE OF GENISTEIN DERIVATIVE IN IMPROVEMENT OF DYSFUNCTION OF LEARNING AND MEMORY AT MULTIPLE TARGETS<br><br>**Int.Class:** A61K 31/453<br><br>**Appl.No:** 201611207108.3<br><br>**Applicant:** INSTITUTE OF MATERIA MEDICA, CHINESE ACADEMY OF MEDICAL SCIENCES<br><br>**Inventor:** LIU AILIN<br><br>Published on 03.07.2018	5-hydroxy-7-O-ethylpiperidine isoflavone (DL0410-3, WS1102001).<br><br>Naturally occurring isoflavone and phytoestrogen mainly found in leguminous plants and particularly enriched in the soybean.<br><br>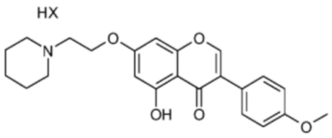<br><br>Patent document	Synthetic Genistein derivative endowed with dual acetylcholinesterase (AChE)/butyrocholinesterase (BuChE) inhibitor with equal affinity to both enzymes. The parent compound, Genistein, is permeable to the BBB and has a multimodal action as an antioxidant, anti-inflammatory, anti-amyloid β, and autophagy promoter (Nazari-Khanamiri and Ghasemnejad-Berenji, 2021; Mas-Bargues et al., 2022).<br><br>The brain permeable parent compound, Genistein, was shown *in vivo* and *in vitro* studies to be neuroprotective in AD through multiple mechanisms, including inhibition of Aβ synthesis, reduction of tau phosphorylation, protection against Aβ-induced apoptosis and oxidative stress (Li et al., 2022b). In addition, Genistein reduced neuroinflammation through inhibition of TLR and activation of PPAR-γ signalling pathways (Valles et al., 2010; Yu et al., 2013). Recently published results from a clinical trial (GENIAL) on nutritional supplementation with genistein, for 12 months in prodromal AD patients, showed amelioration of cognition and prevention of further amyloid deposition (Viña et al., 2022).	*In vivo*, DL0410-3 is said to display low toxicity and good BBB-permeability; additional data reported a reduction of IL-1β and IL-6 and an improvement in cognitive functions when administered to AD animal models (Liu et al., 2018b).<br><br>Notably, a previously developed compound (DL0410) with a similar mechanism of action as a dual AchE/BchE inhibitor, has also been shown to exert neuroprotective and anti-inflammatory activity in AD models (Lian et al., 2017; Zhang et al., 2021c).	Based on its recognized benefits, genistein has attracted attention for the development of a number of derivatives with improved pharmacokinetics and target selectivity, along with potentiated protective features, including DL0410-3 (Fang et al., 2014). Preliminary data suggest that DL0410-3 combines an anti-inflammatory action with the beneficial effects on cholinergic transmission typical of cholinesterase inhibitors, with good inhibition of both AchE and BuChE, setting the ground for future developments of the molecule in AD therapy.

Patent Details	Drug	Mechanism of Action (Neuroinflammation)/grounds for activity in neuroinflammation	Data from preclinical AD models	Comments/Perspectives
IN201647017414: ANTI NEUROINFLAMMATORY AND PROTECTIVE COMPOUNDS IN ACHILLEA FRAGRANTISSIMA<br><br>**Int.Class:** A61K 36/28<br><br>**Appl.No:** 201647017414<br><br>**Applicant:** THE STATE OF ISRAEL MINISTRY OF AGRICULTURE & RURAL DEVELOPMENTAGRICULTURAL RESEARCH ORGANIZATION (ARO) (VOLCANI CENTER).<br><br>**Inventor:** ELMANN Anat<br><br>Published on 31.08.2016	3,4',5-Trihydroxy-3',6,7-trimethoxyflavone (TTF) and achillolid A are naturally-derived compounds extracted from the desert plant *Achillea fragrantissima*<br><br>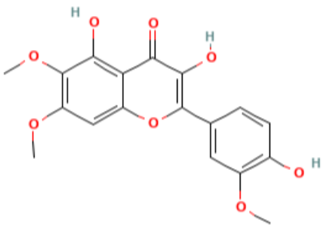<br><br>Pubchem	Both compounds have been shown to provide direct protective effects on neuronal cells due, at least partially, to inhibitory effects on the levels of reactive oxygen species (Elmann et al., 2017; Bartolotti et al., 2018). In addition, TTF was shown to exert anti-oxidant effects on astrocyte cultures through inhibition of a number of key signalling pathways (Elmann et al., 2014). According to the *in vitro* data provided with the patent, both TTF and Achillolid A were able to reduce a number of markers of inflammation and oxidation in LPS-treated microglia, while Aβ effects have not been investigated in this cell type.	In neuronal cultures, TTF prevented the Aβ-induced neuronal death and attenuated the intracellular accumulation of reactive oxygen species (ROS) following treatment with Aβ. TTF also inhibited the Aβ-induced phosphorylation of the signalling proteins SAPK/JNK and ERK 1/2, which belong to the mitogen-activated protein kinase (MAPK) family (Telerman et al, 2017).<br><br>The compounds were also able to contrast the Aβ-induced activation of SAPK/JNK and pERK pathways in neuronal cells (Telerman et al, 2017).	The data of the two compounds effects as anti-inflammatory agents in AD are encouraging but still preliminary. More research needs to be done to better dissect the mechanisms of action, the cellular targets involved and the *in vivo* effects, in order to depict a clearer picture of their true potential.
IN201947028836: BUTYLPHTHALIDE-TELMISARTAN HETEROCOMPLEX PREPARATION METHOD AND APPLICATION THEREOF	Active butylphthalide ring opening butylphthalide-telmisartan hybrid	Telmisartan is an antihypertensive drug that belongs to the class of angiotensin type 1 receptor (AT1R) antagonists. As other drugs in this class, telmisartan is a candidate for repositioning in the treatment of neurological conditions, due to its	Telmisartan inhibited PPARγ-, NF-kB- and IL1β-mediated inflammation *in vitro* and in different *in vivo* models of neuropathologies, including AD (Garrido-Gil et al., 2012; Pang et al., 2012; Xu et al., 2015; Torika et al., 2016; Wang et al., 2020b). In agreement with telmisartan's	This patent includes an association of the two compounds into a new drug, also including a new method for its synthesis. The butylphthalide-telmisartan heterocomplex was designed on the principle of prodrug combination, using the ring-opening derivative of butylphthalide, i.e. potassium 2-(1-hydroxypentyl)-

Patent Details	Drug	Mechanism of Action (Neuroinflammation)/grounds for activity in neuroinflammation	Data from preclinical AD models	Comments/Perspectives
**Int.Class:** C07D 235/18A<br><br>**Appl.No:** 201947028836<br><br>**Applicant:** GUANGDONG LONGFU MEDICINE CO., LTD.<br><br>**Inventor:** HUANG, Zhangjian<br><br>Published on 19.07.2019	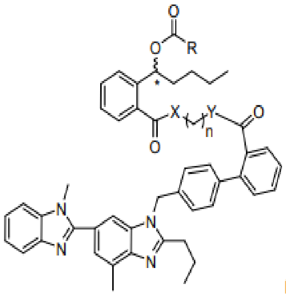<br>Formula I<br><br>Patent document	anti-inflammatory properties (Ihara and Saito, 2020).<br><br>3-N-Butylphthalide (NBP), initially isolated from the seeds of celery (*Apium graveolens Linn*.), is a drug used in the management of stroke, with established neuroprotective effects. NBP was shown to be protective in a number of *in vivo* models of neurodegeneration (Abdoulaye and Guo, 2016). In stroke models, NBP promoted a better post-stroke outcome *in vivo*, with a multitargeted action on several mechanisms, from oxidative stress to mitochondrial dysfunction to apoptosis and inflammation (Liu et al, 2021; Liu et al., 2022b; Zhang et al., 2023).	postulated beneficial role, cortical AT1Rs were shown to be increased in brain areas with ongoing inflammation in AD models (Torika et al., 2016). Efficacy of intranasal administration of the drug in the long term was analysed in 5XFAD mice, where improvements in brain pathology, glial reactivity and cognitive abilities were confirmed (Torika et al., 2017).<br><br>NBP was shown to inhibit key inflammatory pathways, such as NLRP3, in PD (Chen et al., 2018; Que et al., 2021) and NF-κB in AD models *in vitro* and *in vivo*, with effects directed to glial cells (Wang et al., 2013b; Wang et al., 2019). In AD transgenic mice, NBP attenuated astroglial activation, improved cognitive function and reduce Aβ burden (Peng et al., 2010).	benzoate (PHPB). The preparation was shown to possess an improved water-solubility and a doubled oral bioavailability compared to NBP. The repurposing of the two drugs with improved qualities and combinatorial effects directed at key players in inflammation, appears as a valuable approach to pursue.
EP10.3746057: METHOD FOR PREVENTING OR TREATING ALZHEIMER'S DISEASE<br><br>**Int.Class:** A61K 31/10	Dapansutrile (OLT1177), orally active β-sulfonyl nitrile molecule	Dapansutrile is an orally active β-sulfonyl nitrile molecule, previously shown to selectively inhibit the activation of the NLRP3 inflammasome, thereby reducing downstream release of the inflammatory mediators IL-1β and IL-18. Its safety has been assessed in healthy humans (Marchetti et al., 2018a). The efficacy of	The oral administration of depansutrile to transgenic AD mice for 3 months reduced plaque pathology, preserved synaptic plasticity, reduced microglial reactivity and rescued the animals from cognitive impairment, compared to WT mice. In addition, plasma metabolic markers of AD were normalized	The evidence on the safety of depansutrile, together with its ability to selectively block one of the main inflammatory pathways especially relevant in glial cells' activation, are in support of its therapeutic potential. Validation from other laboratories will be required to further strengthen the current evidence.

Patent Details	Drug	Mechanism of Action (Neuroinflammation)/grounds for activity in neuroinflammation	Data from preclinical AD models	Comments/Perspectives
**Appl.No:** 19746676<br><br>**Applicant:** OLATEC THERAPEUTICS LLC<br><br>**Inventor:** DINARELLO CHARLES A<br><br>Published on 09.12.2020	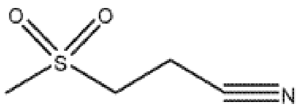<br><br>Patent document	dapansutrile has been shown in a number of studies, all authored by the patent holder, in different animal models of inflammatory conditions. Among these are: joint inflammation (Marchetti et al., 2018b), Experimental Autoimmune Encephalomyelitis (Sánchez-Fernández et al., 2019), spinal cord injury (Amo-Aparicio et al., 2022) and allergic asthma (Lunding et al., 2022). Additionally, dapansutrile is currently under phase 2 clinical investigation for the treatment of gout flares (Klück et al., 2020).	to WT levels in a dose-dependent fashion (Lonnemann et al., 2020).	
EP3802568: PEPTIDE THERAPEUTICS FOR TREATING ALZHEIMER'S DISEASE AND RELATED CONDITIONS<br><br>**Int.Class:** C07K 14/00<br><br>**Appl.No:** 19816181<br><br>**Applicant:** UNIV OKLAHOMA<br><br>**Inventor:** KASUS-JACOBI ANNE<br><br>Published on 14.04.2021	This patent includes three hematopoietic serine proteases found in the azurophilic granules of neutrophils: i) cationic antimicrobial protein of 37 kDa (CAP37), ii) cathepsin G (CG), and iii) neutrophil elastase (NE).<br><br>(Peptide < 40 amino acids)	In addition to their degrading action inside phagolysosomes, these proteases are released during neutrophil activation and contribute to the regulation of inflammatory and immune responses (Korkmaz et al., 2010).	All three peptides have been shown to exert anti-inflammatory activity in AD by targeting two key players of neuroinflammation, i.e. Aβ and the receptor for advanced glycation end-products (RAGE) (Stock et al, 2018). RAGE expression is up-regulated in the brain of patients with AD, where it mediates the toxic effects of Aβ through a direct interaction with the peptide. In microglial cells, RAGE mediates pro-inflammatory activation by interaction different ligands (Yan et al., 2009). Finally, at the BBB, RAGE mediates the influx of circulating Aβ into the brain, and may contribute to endothelial damage (Wan et al., 2014). Upon	Despite the evidence on their beneficial effect against Aβ-triggered inflammation, the peptides have also been implicated in a detrimental role in AD, in connection with their function as mediators of neutrophil activation and would even be responsible for BBB damage and ensuing inflammation (Stock et al, 2018). In agreement, CAP37 has been shown to be upregulated in the brain of AD patients and to recruit and activate microglial cells to release pro-inflammatory mediators (Pereira et al., 1996; Pereira et al., 2003). This critical issue has been reviewed and discussed in a very recent publication (Aries and Hensley-McBain, 2023).

**Patent Details**	**Drug**	**Mechanism of Action (Neuroinflammation)/grounds for activity in neuroinflammation**	**Data from preclinical AD models**	**Comments/Perspectives**
			binding to Aβ, CAP37, CG and NE can operate its cleavage at different sites and with different catalytic activities, resulting in reduced accumulation and fibrillation (Kasus-Jacobi et al., 2021). Furthermore, the peptides can disrupt the Aβ-RAGE interaction both inhibiting downstream inflammatory signalling and preventing Aβ entrance in the brain parenchyma.	In conclusion, the peptide therapeutics presented in this patent are interesting for they combine an anti-amyloid and an anti-inflammatory strategy. This approach is in line with the therapeutical attempts in AD research. The patent describes different administration routes, including intranasal delivery, and suggest the possibility to use appropriate nanocarriers, although no data on the concentration reached in the brain are available. However, central distribution of these peptides could in part mimic the effects of peripheral immune cells infiltration through a BBB, an occurrence that is typical of chronic neuroinflammatory conditions and whose ultimate outcome is still under debate. This surely represents an important issue that will need to be taken into account in future developments.
EP4035669 PREPARATION OF DRUG FOR TREATING ALZHEIMER'S DISEASE<br><br>**Int.Class:** A61K 31/522<br><br>**Appl.No:** 20871356<br><br>**Applicant:** PLANTARX LTD	Combination of acyclovir and dexamethasone	Acyclovir is a guanosine analogue approved in the clinics as an antiviral for the treatment of infections caused by the herpes simplex virus (HSV) (Taylor and Gerriets, 2022). Its potential benefit in AD lays its foundations on the evidence of a link between infections and the risk for AD development. In fact, pathogens can promote the seeding of Aβ by prompting its aggregation to form a barrier around them (Soscia et al., 2010; Piekut et al., 2022). An *in*	The association of acyclovir and DXMT was investigated on the bases of a potential synergy of anti-amyloidogenic effects by acyclovir and anti-inflammatory effects by DMTX. Results showed significantly increased neuroprotective effect of the combination of drugs, compared to single use, against cognitive impairment in mice subjected to intraventricular injection of Aβ oligomers, and 13 days of treatment. The combination	This patent describes an association of acyclovir and dexamethasone and includes successful preclinical data on its effectiveness against cognitive deficits in transgenic AD models.<br><br>Evidence of neuroprotection by GC against dementia appears scarce in the literature (Nerius et al., 2020). Interestingly, it has been recently proposed that the stress-related risk for neurodegeneration is influenced by genetic factors, and that inter-individual differences could impact

Patent Details	Drug	Mechanism of Action (Neuroinflammation)/grounds for activity in neuroinflammation	Data from preclinical AD models	Comments/Perspectives
**Inventor:** CHOI TONY CHUNGLIT<br><br>Published on 03.08.2022		*vitro* study, investigating the relationship between HSV infection, Aβ accumulation, tau phosphorylation and acyclovir treatment, showed a beneficial effect for the drug by a reduction of the viral particles, and consequently Aβ (Wozniak et al., 2011).<br><br>The acyclovir can directly inhibit the activities of indoleamine 2,3-dioxygenase 1 (IDO-1) and tryptophan 2,3-dioxygenase 2 (TDO-2); these two key enzymes are responsible for tryptophan metabolism to prevent quinolinic acid-induced neurotoxicity.<br><br>Dexamethasone (DXMT) is a synthetic glucocorticoid (GC) approved for the treatment of pathologies with allergic, inflammatory or autoimmune components (Johnson et al., 2022). While the anti-inflammatory action of GC would be expected to prove protective against dementia, several *in vitro* and *in vivo* studies have correlated increased levels of GC, such as in chronic stress, to an increased risk of dementia (Green et al., 2006; Vyas et al., 2016; Bisht et al., 2018; Sharma and Singh, 2020; Canet et al., 2022).<br><br>Increased levels of cortisol have been found in AD patients (Ouanes and Popp, 2019; Zheng et al.,	decreased Aβ oligomer-induced microglial and astrocyte activation, pro-inflammatory cytokines upregulation, post synaptic density-95 (PSD95) protein reduction, phospho-Tau expression and spatial cognitive impairment. Notably, DXMT was also shown to contrast mice weight loss that resulted as a side effect of acyclovir treatment (Hui et al., 2020).	the sensitivity to GC stress response and trigger microglia to become pro-inflammatory (Milligan Armstrong et al., 2021).<br><br>The successful results obtained in this preliminary study need to be considered with great caution, in view of the still open debate regarding the true role of GC exposure in AD onset/progression.

**Patent Details**	**Drug**	**Mechanism of Action (Neuroinflammation)/grounds for activity in neuroinflammation**	**Data from preclinical AD models**	**Comments/Perspectives**
		2020b). Targeting of glucocorticoid receptors with selective modulators reduced Aβ levels and rescued the cognitive deficits in transgenic AD mice exposed to early life stress (Lesuis et al., 2018), and chronic exposure to DMTX, to simulate stress responses in AD animal models, negatively affected Aβ and tau pathology, leading to memory deficits (Joshi et al., 2012; Canet et al., 2018).		

Agents targeting key enzymes/receptors involved in potentially detrimental biochemical pathways when deregulated (N=4)

**Patent Details**	**Drug**	**Mechanism of Action (Neuroinflammation)/grounds for activity in neuroinflammation**	**Data from preclinical AD models**	**Comments/Perspectives**
IN201647029441: USE OF FLAP INHIBITORS TO REDUCE NEUROINFLAMMATION MEDIATED INJURY IN THE CENTRAL NERVOUS SYSTEM<br><br>**Int.Class:** A61K 31/470<br><br>**Appl.No:** 201647029441<br><br>**Applicant:** BIOSCIENCE PHARMA PARTNERS, LLC	5-lipoxygenase activating protein (FLAP) inhibitor (a.k.a BPP 1001)<br>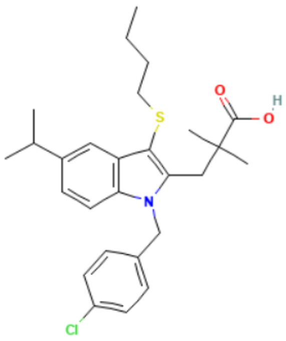<br>MK-866 – Pubchem	FLAP is the rate limiting enzyme in the biosynthesis of leukotrienes (Peters-Golden and Brock, 2003). Cysteinyl leukotrienes (CLTs) have been involved in a broader range of pathologies characterized by inflammation, including vascular inflammation, cancer and a number of CNS disorders like multiple sclerosis, AD, Parkinson's disease, brain ischemia and epilepsy (Ghosh et al., 2016; He et al., 2020). FLAP levels have been observed to increase with age in vulnerable brain areas such as the hippocampus (Chu et al., 2013) and FLAP polymorphisms were linked	Recently, FLAP was shown to be expressed only by microglial cells in the brains of both transgenic AD mice and AD patients, and microglial depletion negatively affected both FLAP and 5-LO levels (Michael et al., 2020).<br><br>On these bases, blockade of CLTs signalling has been proposed as a therapeutic approach.<br><br>Administration of FLAP inhibitor MK-591 reduced Aβ synthesis and tau phosphorylation and decreased GSK-3β activity, in an in vivo AD mouse model (Chu and Praticò, 2012; Chu et al.,	The preclinical evidence on the benefits of FLAP inhibitors in AD so far encourages their development. The data made available within the patent documents relate exclusively to the effects of compounds MK-886 and MK-591 in a model of traumatic brain injury ((Heidenreich and Murphy, 2016), with no further information on the potential benefit in AD.<br><br>The possibility, reported in the patent, for nasal delivery, a route that avoids BBB, is an additional advantage to the use of this class of drugs.

Patent Details	Drug	Mechanism of Action (Neuroinflammation)/grounds for activity in neuroinflammation	Data from preclinical AD models	Comments/Perspectives
**Inventor:** HEIDENREICH, KiM,<br><br>Published on 07.10.2016	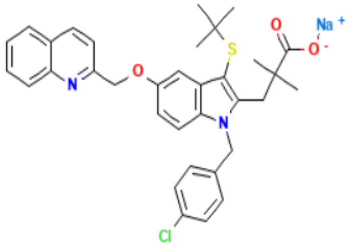<br>MK-591– Pubchem	to vascular pathology in AD (Manev and Manev, 2006).<br><br>In the patent document, administration of MK-886 in a model of traumatic brain injury blocked injury-induced LTD4 synthesis, reduced cell death, attenuated brain oedema and deficits in hippocampal long-term potentiation and decreased the impairment of memory and learning (Heidenreich and Murphy, 2016). Less extensive, data in the same model, were also presented for MK-591.	2013). These data were confirmed in a different mouse model of AD, where blockade of FLAP by MK-591 was also associated with cognitive improvement and synaptic protection at early stages (Giannopoulos et al., 2013). Additionally, MK-886 was shown to reduce PPAR-γ and plaque load in AD mice (Hawkes et al., 2014)	
KR1020180036318: COMPOSITION FOR PREVENTING OR TREATING NEURODEGENERATIVE DISEASES AND DEPRESSION COMPRISING DERIVATIVE OF 2-AMINO-2-(1-DODECYL-1H-1,2,3-TRIAZOL-4-YL) PROPANE-1,3-DIOL AS ACTIVE INGREDIENT<br><br>**Int.Class:** A61K 31/4192	Derivative of 2-amino-2-(1-dodecyl-1h-1,2,3-triazol-4-yl)propane-1,3-diol<br>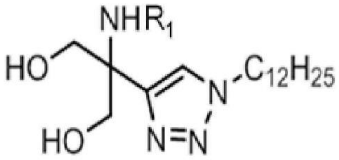<br>Patent document	The active ingredient included in this patent is endowed with acid sphingomyelinase (ASM) inhibitor activity. ASM converts sphingomyelin to ceramide. This pathway can be activated as a cellular stress response, involving signalling molecules like death ligands, pro-inflammatory cytokines and cytotoxic agents, thus playing a key role in inflammation (Zeidan and Hannun, 2010; Henry et al., 2013; Bienias et al., 2016). ASM has been shown to mediate BBB disruption in aging, an occurrence that can intensify central inflammation due to barrier leakage (Park et al., 2019).	Data from AD animal models, made available with the patent, showed reduced ASM plasma and brain levels, reduced amyloid pathology, astroglial reactivity and pro-inflammatory cytokine expression (Bae et al., 2018). Of note, ASM inhibitors have also been shown to possess antidepressant effects and, in agreement, antidepressant drugs have been shown to possess anti-ASM activity (Gulbins et al., 2013; Yang et al., 2020). The antidepressant effects have also been observed with the patented active ingredient (Bae et al., 2018).	Dysregulation of sphingolipid metabolism has been associated with a number of pathological states, including neuroinflammatory-driven diseases (Pralhada Rao., et al 2013). The active ingredient included in this patent represents a novel ASM inhibiting compound with improved efficacy compared to the ASM inhibiting antidepressant drug amitriptyline. The proposed dual activity of this compound on both Aβ burden, and neuroinflammation as well as depression, is interesting although at present no published data are available.

**Patent Details**	**Drug**	**Mechanism of Action (Neuroinflammation)/grounds for activity in neuroinflammation**	**Data from preclinical AD models**	**Comments/Perspectives**
**Appl.No**: 1020160126750<br><br>**Applicant:** KYUNGPOOK NAT UNIV IND ACADEMIC COOP FOUND<br><br>**Inventor**: BAE, JAE SUNG<br><br>Published on 09.04.2018		Several studies report abnormal sphingolipid metabolism in AD, where ASM and ceramide levels are increased in the brain of patients, already evident in early stages of disease progression (Katsel et al., 2007; He et al., 2010; Filippov et al., 2012). Aberrant ceramide expression was shown selectively in astroglial cells in AD brains (Satoi et al., 2005).<br><br>Also, impaired autophagic processes were rescued by ASM inhibition in AD mouse models, with improved Aβ clearance and a positive outcome on cognition (Lee et al., 2014).		
CN106924246: APPLICATION OF ISOFLAVONE COMPOUND TO PREVENTING OR TREATING NEURODEGENERATIVE DISEASES<br><br>**Int.Class:** A61K 31/4025<br><br>**Appl.No:** 201611266702.X<br><br>**Applicant:** INSTITUTE OF MATERIA	Isoflavone compound J37941<br><br>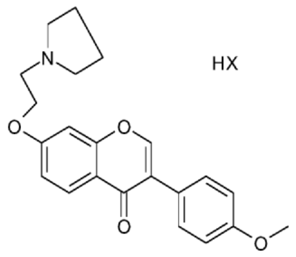<br><br>Patent document	Isoflavones are substituted derivatives of isoflavone, and are phytoestrogens that originate almost exclusively from the soybean and other leguminous plants (Křížová et al., 2019).<br><br>The patent claims that the isoflavone compound J37941 inhibits AchE and BuChE dose-dependently, thereby exerting a neuroprotective action against oxidative stress and inflammation.	According to the patent, J37941 was shown to be BBB permeable, and it displayed relatively low toxicity in vivo. Furthermore, J37941-induced cognitive improvement has been reported in a mouse model of dementia caused by scopolamine (Wang et al., 2017b).	The mechanism of action of this drug is not innovative, but its dual action on both esterases and the improved pharmacokinetics might make it a candidate worth further investigation.

Patent Details	Drug	Mechanism of Action (Neuroinflammation)/grounds for activity in neuroinflammation	Data from preclinical AD models	Comments/Perspectives
MEDICA, CHINESE ACADEMY OF MEDICAL SCIENCES<br><br>**Inventor:** LIU AILIN<br><br>Published on 07.07.2017				
EP3806956: METHODS FOR PREVENTING OR DELAYING ONSET OF ALZHEIMER'S DISEASE AND OTHER FORMS OF DEMENTIA AND MILD COGNITIVE IMPAIRMENT<br><br>**Int.Class:** A61P 25/00<br><br>**Appl.No:** 19819383<br><br>**Applicant:** DANDREA MICHAEL R<br><br>**Inventor:** D'ANDREA MICHAEL R<br><br>Published on 21.04.2021	Alpha 7 acetylcholine nicotine receptor binding agents (the following are mentioned in patent documents; alpha-bungarotoxin, Nicotine, Varenicline, GTS-21, Methyllycaconitine)	The α7 acetylcholine nicotine receptor (A7R) is widely expressed in neuronal, glial and endothelial cells, where its activation has been shown to regulate both physiological and pathological processes, likely depending on the cell type and microenvironmental context (extensively reviewed in Xu et al., 2021b).	Aβ(1-42) has been shown to bind to neuronal A7R with high affinity. The resulting complex is internalized by the cell, with the involvement of p38 signalling (Ma et al., 2018), and can ultimately lead to synaptic impairment and cell death, accounting for selective Aβ-vulnerability of A7R-expressing cholinergic neurons in AD brains (Wang et al., 2000; Nagele et al., 2002; Farhat and Ahmed, 2017; Ma et al., 2018). At the surface of endothelial cells, the A7R-Aβ complex has been proposed to disrupt BBB integrity and to contribute to abnormal circulating Aβ entry in the brain (Liu et al., 2017). The targeting of the alpha 7 nicotinic acetylcholine receptor has been described to reduce amyloid accumulation in Alzheimer's disease pyramidal neurons by D'Andrea and Nagele (D'Andrea and Nagele, 2006).<br><br>Varenicicline, an A7R agonist used for smoke cessation, has	The patent holder's primary target is reported to be the prevention of A7R-Aβ complex-mediated BBB disruption by administration of A7R binding agents. Treatment is proposed to prevent the excessive accumulation of bloodstream-derived Aβ in the CNS and the detrimental consequences on neuronal cells. The specific A7R binding agents can be either novel or re-purposed, and include agonists, antagonists, inhibitors and allosteric modulators and are meant as an adjunctive therapy together with other therapeutical approaches, with a selection of the potential target population by analysis of appropriate biomarkers, especially those indicative of a BBB impairment (D'Andrea, 2021).

Patent Details	Drug	Mechanism of Action (Neuroinflammation)/grounds for activity in neuroinflammation	Data from preclinical AD models	Comments/Perspectives
			recently entered a phase II cross-over study in Korea, but results showed no improvement in cognitive and behavioural outcomes in the analysed population (Kim et al., 2014).	

Compounds still in need of clarification (N=1)

Patent Details	Drug	Mechanism of Action (Neuroinflammation)/grounds for activity in neuroinflammation	Data from preclinical AD models	Comments/Perspectives
EP1478634: FURANONE DERIVATIVES<br><br>**Int.Class:** A61K 31/34<br><br>**Appl.No:** 03705988<br><br>**Applicant:** GALILEO PHARMACEUTICALS INC<br><br>**Inventor:** WANG BING<br><br>Published on 24.11.2004	FURANONE DERIVATIVES especially 3-hydroxy-furan-2 one-derivatives (several compounds are described in this patent). Small Molecule<br>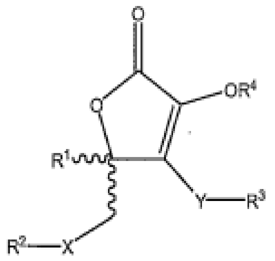<br>General Structure – patent document	Furanones are heterocyclic compounds whose many derivatives have found application as regulators of several biological processes, including inflammation (Husain et al., 2019).	Selective studies on the exact mechanism of action and targeted pathways of the patented furanone derivatives in AD are not currently available.	This patent includes a number of furanone derivatives, developed as claimed anti-inflammatory protective agents, and potentially useful for the treatment of neurodegenerative disorders, including Alzheimer's disease, stroke, cerebral ischemia, and other oxidative stress–related conditions (Wang et al., 2004). The lack of available information from pre-clinical studies prevents at present any evaluation.

Abbreviations: AD-Alzheimer Disease, ADA-Anti-drug antibodies, ADAS-Cog-Alzheimer's Disease Assessment Scale-Cognitive subscale, ADCOMS-Alzheimer's Disease Composite Score, ADCS CGIC; Alzheimer's Disease Cooperative Study Clinical Global Impression of Change, ADCS-ADL-Alzheimer's Disease Cooperative Study Activities of Daily Living Scale, ADCS-ADL-MCI-Alzheimer's disease co-operative study activities of daily living scale for mild cognitive impairment, ADCS-CGIC-Alzheimer's Disease Cooperative Study - Clinical Global Impression of Change, AEs-Adverse Events, ApoE-apolipoprotein E, AUC-Area Under Curve, Aβ-Amyloid beta, BBB-Blood Brain Barrier, BOLD-blood-oxygen level depended, CCL2-chemokine (C-C motif) ligand 2, CDR-Clinical Dementia Rating, CDR-SB-Clinical Dementia Rating scale Sum of Boxes, CIBIC-Clinician's Interview-Based Impression of Change, CL-Total Clearance, C_{max}-Maximum Observed Plasma Concentration, CSF-Cerebrospinal Fluid, CSF-1R-Colony-stimulating factor-1 receptor, C-SSRS-Columbia Suicide Severity Rating Scale, C_{trough}-Concentration at the End of a Dosing Interval, DMI-Delayed Memory Index, DSST-Digit

symbol substitution test, DTI-NODDI-Diffusion Tensor Imaging - Neurite Orientation Dispersion Density Imaging, eCOG-Everyday Cognition, EMACC-Early and Mild Alzheimer's Cognitive Composite, EQ 5D 5L QoL - EuroQol 5 Dimension 5-Level quality of life, ESR-Erythrocyte Sedimentation Rate, FDG PET-Fluorodeoxyglucose Positron Emission Tomography, fMRI-functional Magnetic Resonance Imaging, GDS-Global Deterioration Scale, GM-CSF-Granulocyte macrophage colony-stimulating factor, HVLT-R-Hopkin's verbal learning task-revised, IL-Interleukin, MAPK-mitogen-activated protein kinase, MCI-Mild Cognitive Impairment, MCP-1-Monocyte chemoattractant protein-1, MERET OBSRO-C-Memory-Enhanced Retrospective Evaluation of Treatment Observer Reported Global Impression of Improvement, MERET PGI-C-Memory-Enhanced Retrospective Evaluation of Change from baseline Global Impression of Improvement, MMSE-Mini-Mental State Exam, MoCA-Montreal Cognitive Assessment, MRI-Magnetic Resonance Imaging, MRS-Magnetic Resonance Spectroscopy, NfL-neurofilament light chain protein, NF-κB-Nuclear factor kappa B, NIA-AA-National Institute on Aging Alzheimer's Association, NINCDS-ADRDA-National Institute of Neurological and Communicative Disorders and Stroke - Alzheimer's Disease and Related Disorders Association, NPI-Neuropsychiatric Inventory, NPI-Q-Neuropsychiatric Inventory Questionnaire, NVR-Neurovascular Coupling, PD-Pharmacodynamics, PDE5-phosphodiesterase 5, PET-Positron Emission Tomography, PK-Pharmacokinetics , RBANS-Repeatable Battery for the Assessment of Neuropsychological Status, SAGE-Self-Administered Gerocognitive Exam, SD-standard deviation, SIB-Severe Impairment Battery, t(1/2)-Terminal Elimination Phase Half-life, TEAE-Treatment-emergent Adverse Events, TGF-β1-Transforming Growth Factor-β1, T_{max}-Time at which Maximum Plasma Concentration Occurs, TNFα-Tumour Necrosis Factor alpha, TREM2-Triggering receptor expressed on myeloid cells 2, TSPO-Translocator Protein, V_d-Volume of distribution.
